# Assessing Potential Factors Influencing the Efficacy of Immune Checkpoint Inhibitors with Radiation in Advanced Non-Small-Cell Lung Cancer Patients: A Systematic Review and Meta-Analysis

**DOI:** 10.1155/2023/4477263

**Published:** 2023-01-13

**Authors:** Huilin Xu, Dedong Cao, Dingjie Zhou, Anbing He, Wei Ge, Ximing Xu

**Affiliations:** ^1^Department of Oncology, The Fifth Hospital of Wuhan, Wuhan, Hubei 430000, China; ^2^Department of Oncology, RenMin Hospital of Wuhan University, Wuhan, Hubei 430000, China; ^3^Department of Oncology, Taikang Tongji (Wuhan) Hospital, Wuhan, Hubei 430000, China

## Abstract

**Objective:**

Recent evidence suggests that combining radiotherapy (RT) with immune checkpoint inhibitors (ICIs) may result in better outcomes. In this study, we assessed the efficacy and safety of ICI plus radiation versus ICI alone and explored potential factors affecting its efficacy in advanced non-small-cell lung cancer (NSCLC) patients.

**Methods:**

The databases including PubMed and Embase were searched to retrieve eligible studies comparing the efficacy and safety outcomes in advanced NSCLC patients after ICIs ± RT treatments. We performed subgroup analyses to identify potential prognostic factors from radiation details and study types. The odds ratio (OR) of objective response rate (ORR) and disease control rate (DCR), hazard ratio (HR) of progression-free survival (PFS) and overall survival (OS), and risk ratio (RR) of adverse events were used to represent the outcome effects.

**Results:**

26 eligible studies with 14192 cases were included. The results showed that the ORR (OR = 0.63, 95% CI: 0.42, 0.93; *p* = 0.02) and DCR (OR = 0.55, 95% CI: 0.36, 0.82; *p* < 0.01) of RT + ICIs groups were significantly higher than those of the ICIs alone group. The median PFS and OS for ICIs versus RT + ICIs were 2.2 versus 4.4 months and 9.0 versus 13.4 months, respectively. Patients in the ICIs plus RT group had a significantly better PFS (HR = 0.72, 95% CI: 0.64, 0.81; *p* < 0.01) and OS (HR = 0.74, 95% CI: 0.65, 0.83; *p* < 0.01) when compared to those in the ICIs group. In terms of adverse events, the risk of pneumonia was not significantly increased in patients treated with both ICIs and RT when compared to ICIs group alone (risk ratio = 0.89; 95% CI: 0.55, 1.44; *p* = 0.63). The correlation analysis found that PFS was significantly correlated with OS (p = 0.02). The subgroup analysis results showed that significant improvements in OS were observed in non-palliative RT group (HR = 0.29, 95% CI: 0.13, 0.65; *p* < 0.01) and extracranial RT group (HR = 0.70, 95% CI: 0.59, 0.83; *p* < 0.01). RT type could also be a prognostic factor associated with the OS (for conventional RT: HR = 0.68 and *p* = 0.22; for stereotactic body radiation therapy: HR = 0.77 and *p* < 0.01). However, concerning RT timing, the results showed a similar trend in reducing mortality risk (for previous RT: HR = 0.64 and *p* = 0.21; for concurrent RT: HR = 0.35 and *p* = 0.16).

**Conclusion:**

RT plus ICIs is associated with improved survival for advanced NSCLC patients, especially for those with non-palliative RT. Further clinical trials are needed to validate its effect on survival outcomes.

## 1. Introduction

Lung cancer is one of the most common causes of cancer deaths worldwide [1]. Non-small-cell lung cancer (NSCLC) is the most common subtype of lung cancer, accounting for about 76% of all lung cancers [1]. In recent years, based on the understanding of tumor biology and the mechanism of occurrence and development, important progress has been achieved in the treatment of advanced NSCLC [2, 3]. Among them, immune checkpoint inhibitors (ICIs) are considered one of the most promising agents [4, 5]. Although the clinical administration of this therapy can bring significant efficacy and survival improvement for these patients, its efficacy is closely related to the expression of PD-L1 [3], tumor mutation burden [6], neutrophil/lymphocyte ratio [7], and body mass index [8]. There are still a considerable number of patients who fail to benefit from immunotherapy due to factors such as programmed death-ligand 1 (PD-L1) expression [3], epidermal growth factor receptor (EGFR) mutation status [9], low tumor mutation burden (TMB) [6], high neutrophil-lymphocyte ratio(NLR) [7], or low body mass index (BMI) [8]. Therefore, how to improve the efficacy of ICIs in advanced NSCLC is still under investigation.

The key to improving the antitumor efficacy of immune checkpoint inhibitors lies in combination therapy [10–13]. Radiotherapy, chemotherapy, molecular targeted therapy, and other cancer treatment methods can have a synergistic effect to improve the efficacy of immunotherapy [10–13]. Studies have found that radiotherapy can enhance the efficacy of immunotherapy in locally advanced NSCLC [11, 14]. The PACIFIC study included 713 patients with locally advanced inoperable NSCLC [14]. The median progression-free survival (PFS) of the group receiving durvalumab consolidation therapy after concurrent radiotherapy and chemotherapy was 17.20 months, while that of the control group was only 5.60 months (HR = 0.68 and *p* < 0.01); the median overall survival (OS) was 47.50 months, and that of the control group was 29.10 months (HR = 0.71 and *p* < 0.05) [14].

Due to the limitation of ICIs monotherapy, the application of radiotherapy combined with immunotherapy in advanced NSCLC is increasing in recent years, with palliative or non-palliative intents. However, the improvement in survival is not consistent. This raises several concerns about the factors affecting the efficacy of this combination strategy [15–19]. Besides, there are studies that fail to support that adding RT to ICIs can improve treatment outcomes when combining ICIs with RT [20–23]. A study screened 121 patients diagnosed with metastatic NSCLC and compared OS and PFS for patients with ICIs ± RT. The results showed that no difference was found between patients receiving ICIs versus ICIs + RT in terms of median OS (16.7 months versus 16.2 months, *p* > 0.05) or PFS (9.3 months versus 10.7 months, *p* > 0.05). They concluded that the use of RT in addition to ICIs was not associated with improved OS or PFS in metastatic NSCLC patients [23]. Whether adding RT to ICIs could result in a significantly higher risk of adverse events is also not known.

Considering there are still inconsistent results about the efficacy and safety of combination therapy in advanced NSCLC and the concerns about factors affecting survival, we performed this systematic review and meta-analysis. By searching the relevant clinical studies of radiotherapy combined with immunotherapy for advanced NSCLC patients, we evaluated the efficacy and safety of RT + ICIs versus ICIs and explored the influence of RT details, such as timing, sites, and types of radiotherapy, on the treatment outcomes in patients with advanced NSCLC by subgroup analyses.

## 2. Methods

This study was registered in PROSPERO (https://www.crd.york.ac.uk/prospero/) (#CRD42019120007). This study was performed according to the Preferred Reporting Items for Systematic Reviews and Meta-Analyses (PRISMA) checklist [24] (Supplemental Table 1).

### 2.1. Search Strategy

The PubMed and Embase databases were searched to identify eligible studies comparing the efficacy and safety outcomes in advanced NSCLC patients treated with ICIs versus RT + ICIs. The preprint platforms, such as bioRxiv, were also searched to retrieve unpublished studies on the same topic mentioned above. References of important reviews were also identified to further include eligible studies. The search was conducted in the above databases until August 2022. No language limitation was applied during the search. The search terms were as follows: “non-small cell lung cancer,” “NSCLC,” “radiotherapy,” “stereotactic body radiation therapy (SBRT),” “SBRT,” “stereotactic ablative radiotherapy,” “immunotherapy,” “immune checkpoint inhibitor,” “nivolumab,” “pembrolizumab,” “anti-pd-1,” “pd-1 inhibitor,” “durvalumab,” “atezolizumab,” and “cytotoxic T lymphocyte-associatedantigen-4inhibitor.” The search terms were used in different combinations and were adjusted according to specific databases. The example of the search strategy is presented in Supplemental Table 2.

### 2.2. Inclusion and Exclusion Criteria

Inclusion criteria were designed based on the previously registered protocol (#CRD42019120007) in “https://www.crd.york.ac.uk/PROSPERO/.” The inclusion criteria were as follows: study types—retrospective or prospective studies; patients—subjects with a confirmed diagnosis of advanced/metastatic NSCLC by pathology or cytology; and interventions—ICIs were used with or without RT. RT was administrated according to the treatment goals described in the included studies. Comparisons of treatment effectiveness of ICIs versus RT + ICIs were reported in the eligible studies. Outcome indicators: these included ORR (overall response rate), DCR (disease control rate), PFS, OS, safety, and prognostic factors related to RT. Subgroup: subgroup analysis was introduced according to RT and study details. Its details are presented in the statistical methods section. The definitions of main outcome measures are detailed in a previously published study [4]. Animal experiments, duplicate publications, and literature with insufficient data were excluded. References such as comments and review articles were also excluded.

### 2.3. Literature Screening and Data Extraction

Two reviewers (Dedong Cao and Dingjie Zhou) conducted the literature search, read the titles and abstracts of studies after the preliminary search, and then read the full text of eligible studies for further identification, independently. The inconsistencies about the screening results were discussed with a third reviewer (Huilin Xu). The data were extracted by three reviewers (Huilin Xu, Dedong Cao, and Dingjie Zhou), and the extracted information was as follows: (1) basic information of the studies, including the title, the first author, region, the year of publication, age, and sex; (2) cancer-related details, including the number of patients in each group and cancer stage; and (3) treatment details, including the timing of RT (before ICIs, concurrent with ICIs, and multiple time points), types of RT (conventional RT (CRT) and SBRT), and ICI details. Outcome data include the total number of patients, ORR, DCR, PFS, OS, and safety. The reviewers (Dedong Cao and Huilin Xu) checked the extracted data with each other. Disagreements were resolved by re-examining the studies.

### 2.4. Quality Evaluation

For RCT studies, the methods of Cochrane Handbook 5.1 were used to evaluate the quality of the included studies [25]. Five major aspects of bias, such as random, allocation, blinding, selective reporting, and other bias, were included in the assessment. For prospective and retrospective studies, literature quality evaluation was performed according to the Newcastle-Ottawa Scale (NOS) criteria [26], which included three major aspects: selection, comparability, and outcome. After the evaluation, a score ranging from 0 to 9 was calculated for each included study. For a study with six or higher stars, the quality was regarded as high.

### 2.5. Statistical Methods

Meta-analysis was processed using the Comprehensive Meta-Analysis v3.0 software (Biostat Inc.) and RevMan 5.4. The statistical methods of the meta-analysis were introduced as previously reported [27–29]. In brief, the odds ratio (OR) or risk ratio (RR) and its 95% confidence interval (CI) were used for the comparison of dichotomous data. The median and its related 95% CI were used for continuous data. The hazard ratio (HR) and its related 95% CI were used to present the survival benefits from treatments. For OR, RR, or HR, it was considered that the combination group had a better effect if the value was less than 1. The chi-square test and *I*^2^ were used for statistical heterogeneity analysis. According to the Cochrane Handbook, four levels of heterogeneity are classified [25]. If *I*^2^ is below 40%, it indicates that heterogeneity may not be important. *I*^2^ between 30% and 60% indicates moderate heterogeneity, 50%–90% indicates significant heterogeneity, and 75%–100% indicates a greater significant heterogeneity. For analysis with low and moderate risk of heterogeneity, the fixed-effect model is used. If a significant heterogeneity is detected (*I*^2^ > 50% and *p* < 0.1), the source of the heterogeneity will be analyzed and studies that are responsible for this difference will be excluded or subgroup analysis or sensitivity analysis will be performed. In addition, a random-effect model will be used for the meta-analysis. For insufficient data or significant heterogeneity, a descriptive analysis will be performed.

For the continuous data, the median/mean and its confidence interval were extracted as reported in the included studies. The meta-analysis of median variables was performed using the methods as reported [27, 29]. In addition, the median could be regarded as an estimate of the mean if the distribution of the data is symmetrical and therefore be used directly in the meta-analysis [25]. Also, the *p* value of the comparison between ICIs and RT + ICIs was either extracted as reported or calculated using the Review Manager 5.4.1 tool.

### 2.6. Subgroup Setting

The primary endpoints of this meta-analysis were efficacy, survival, and safety of ICIs versus RT + ICIs in advanced NSCLC patients. Additional analyses were also conducted to evaluate the influence of several factors related to RT on the outcomes of RT + ICIs versus ICIs. Subgroup analyses were used to assess the impact of study design (prospective and retrospective), disease condition (advanced and metastatic), and RT timing (prior, concurrent, and multiple) on the efficacy, survival, and safety outcomes. CRT referred to conventional radiotherapy, and SBRT was defined as delivering high-dose radiation (3 or higher Gy per fraction) to eliminate tumors in fewer treatment fractions than CRT. The timing “prior” was defined as delivering RT before ICIs, “concurrent” was defined as RT during the treatment cycles of ICIs, and “multiple” was defined as RT used before, during, and/or after administration of ICIs. The cumulative analysis was used to detect the dynamic trend of meta-analysis and evaluate the impact of a single study on the overall outcomes. The sensitivity analysis was used to evaluate the reliability and stability of the pooled effect by removing one study each time. To detect publication bias, the funnel plot was drawn and Egger's test [25] was used if possible. The cumulative meta-analysis is a sequence of meta-analyses, starting by analyzing a single study at the beginning and adding the rest of the included studies one by one to the analysis until all of them are included in the analysis [30]. It shows the dynamic trends of the overall estimate when adding every single study to the meta-analysis [30]. *p* < 0.05 was considered as there was statistical significance.

## 3. Results

### 3.1. Literature Screening Results

**Figure 1:**
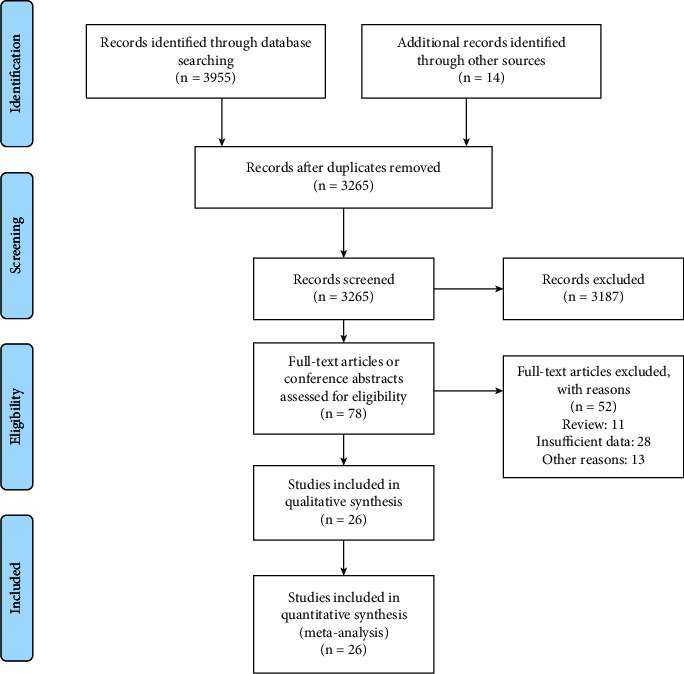
The PRISMA flow diagram of literature screening process and results.

The preliminary search found a total of 3969 references. After excluding duplication and reviewing the title and abstract of the studies, 3187 studies were discarded and the remaining 78 articles were initially included for further identification. Among them, a total of 26 studies [15, 16, 18, 20–23, 31–49] were finally included in the systematic review and meta-analysis after reviewing the full text, with 14192 participants. The literature screening process and results are shown in Figure 1.

### 3.2. Basic Characteristics and Methodological Quality of the Literature

The basic characteristics of the included studies are shown in Table 1. Most of the included studies were retrospective studies, and only 4 prospective studies and 4 RCTs were included. These studies were mainly from America (*n* = 12), Australia (*n* = 3), Europe (*n* = 4), and Asia (*n* = 4). The mean age of the involved patients varied across included studies. All studies reported the treatment strategy, and 8 out of 26 described that SBRT was used to treat cancer. These studies described the treatment line, and 15 of them used the treatment in the ≥ 1st line setting and 11 of them in the ≥2nd line setting.. Studies were grouped into prior (*n* = 15), concurrent (*n* = 6), and multiple (*n* = 5) based on the timing of RT. All studies reported the diagnosis and stage of NSCLC.

The overall quality of the included studies was moderate. Only a few studies described methods of selecting and reporting patients and other sources of biases. In terms of NOS, eight studies were assigned with seven stars, ten with eight stars, and four with nine stars (Supplemental Table 3). The main limitations were loss to follow-up rate and inadequate follow-up. According to the Cochrane Handbook methods, the most common bias was random bias and blinding bias (Supplemental Table 4).

### 3.3. Results of the Main Meta-Analysis

#### 3.3.1. ORR

The ORR was reported in 10 studies. After combining data from the individual studies, the ORRs were 21.9% (218/994) versus 29.8% (158/530) for ICIs versus RT + ICIs groups, respectively. The OR for ICIs versus RT + ICIs was 0.63 (95%CI: 0.42, 0.93; *p* = 0.02), and it was statistically significant.

Subgroup analyses based on study type, disease stage, and RT timing were also performed (Supplemental Figure 1). For prospective and retrospective design, the OR of RT + ICIs versus ICIs was 0.82 (95% CI: 0.26, 2.62; *p* = 0.74) and 0.61 (95% CI: 0.40, 0.92; *p* = 0.02), respectively (Supplemental Figure 1A). With regard to disease types, the OR for comparing ORR of ICIs versus conventional RT (CRT) + ICIs was 0.58 (95% CI: 0.35, 0.96; *p* = 0.04) in the advanced disease group, and it was 0.64 (95% CI: 0.41, 0.99; *p* < 0.05) for the metastatic group (Supplemental Figure 1B). In terms of RT timing, the results showed that when adding radiotherapy concurrently with ICIs, the OR for ICIs versus RT + ICIs was 1.31 (95% CI: 0.19, 8.88; *p* = 0.28). When used radiotherapy before ICIs, the OR for ICIs versus RT + ICIs groups was 0.58 (95% CI: 0.38, 0.89; *p* = 0.01), and it was statistically significant (Supplemental Figure 1C). For patients treated with CRT, the OR for ICIs versus RT + ICIs was 0.71 (95% CI: 0.49, 1.04; *p* = 0.08), while it was 0.40 (95% CI: 0.16, 1.03; *p* = 0.06) for patients with SBRT (Supplemental Figure 2A). For patients that received ≥ first-line treatment, the OR for ICIs versus RT + ICIs was 0.65 (95% CI: 0.47, 0.91; *p* = 0.01), while it was 0.73 (95% CI: 0.36, 1.50; *p* = 0.40) for patients that received ≥ second-line treatment (Supplemental Figure 2B).

#### 3.3.2. DCR

The overall percentages of disease control in ICIs versus RT + ICIs groups were 54.2% versus 62.3% (OR = 0.55, 95% CI: 0.36, 0.82; *p* < 0.01), respectively. It was suggested that RT plus ICIs could provide a significantly better DCR in advanced NSCLC patients.

Subgroup methods were also performed as those of the ORR. For study types, the OR for RT + ICIs versus ICIs was 0.44 (95% CI: 0.23, 0.83; *p* = 0.01) in the prospective studies, and it was 0.63 (95% CI: 0.38, 1.07; *p* = 0.09) in the retrospective studies (Supplemental Figure 3A). With regard to disease condition, adding RT to ICIs resulted in a significantly better DCR than ICIs alone (Supplemental Figure 3B), both in advanced group (OR = 0.63, 95% CI: 0.42, 0.94; *p* = 0.03) and metastatic group (OR = 0.38, 95% CI: 0.15, 0.95; *p* = 0.04). For RT used prior to ICIs, the OR was 0.59 (95% CI: 0.40, 0.86; *p* < 0.01) for ICIs versus RT + ICIs groups (Supplemental Figure 3C). For patients treated with CRT, the OR for ICIs versus RT + ICIs was 0.65 (95% CI: 0.50, 0.84; *p* = 0.001), while it was 0.56 (95% CI: 0.22, 1.44; *p* = 0.23) for patients with SBRT (Supplemental Figure 4A). For patients that received ≥ first-line treatment, the OR for ICIs versus RT + ICIs was 0.81 (95% CI: 0.47, 1.39; *p* = 0.01), while it was 0.49 (95% CI: 0.34, 0.71; *p* < 0.001) for patients that received ≥ second-line treatment (Supplemental Figure 4B).

### 3.4. Survival Endpoints

#### 3.4.1. Survival Summary

**Figure 2:**
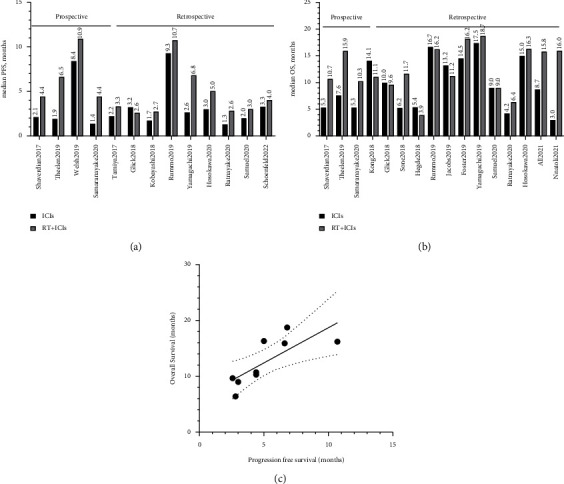
Summary and correlation analysis of PFS and OS in advanced NSCLC patients treated with ICIs versus RT + ICIs regimen. (a) The summarized PFS. (b) The summarized OS. (c) The correlation analysis between PFS and OS from RT + ICIs group.

The reported PFS and OS from the included studies are summarized in Figures 2(a) and 2(b). After analyzing the data from individual studies, the median PFS for ICIs and RT + ICIs groups was 2.2 (95% CI: 1.9, 3.4) and 4.4 (95% CI: 3.3, 6.6) months, respectively. The median OS for ICIs and RT + ICIs groups was 9.0 (95% CI: 5.5, 14.3) and 13.4 (95% CI: 10.5, 16.2) months, respectively. For prospective studies, the PFS and OS were 2.1 (95% CI: 1.7, 5.9) and 5.3 (95% CI: 5.3, 7.6) months for ICIs, while they were 6.4 (95% CI: 4.3, 9.6) and 10.7 (95% CI: 10.3, 15.9) months for RT + ICIs, respectively. For retrospective studies, the PFS was 2.4 (95% CI: 1.7, 3.2) and 3.2 (95% CI: 2.7, 6.7) months, and OS was 11.5 (95% CI: 6.1, 14.8) and 14.6 (95% CI: 10.1, 16.8) months for ICIs versus RT + ICIs groups, respectively.

#### 3.4.2. Correlation Analysis of PFS and OS

To explore the association between PFS and OS, we performed a correlation analysis. A total of 9 pairs of PFS and OS from the RT + ICIs group were included. The tabular result showed that the correlation Pearson *r* value was 0.77 (95% CI: 0.21, 0.95; *p* = 0.02), with a squared *R* of 0.59 (Figure 2(c)).

#### 3.4.3. PFS

**Figure 3:**
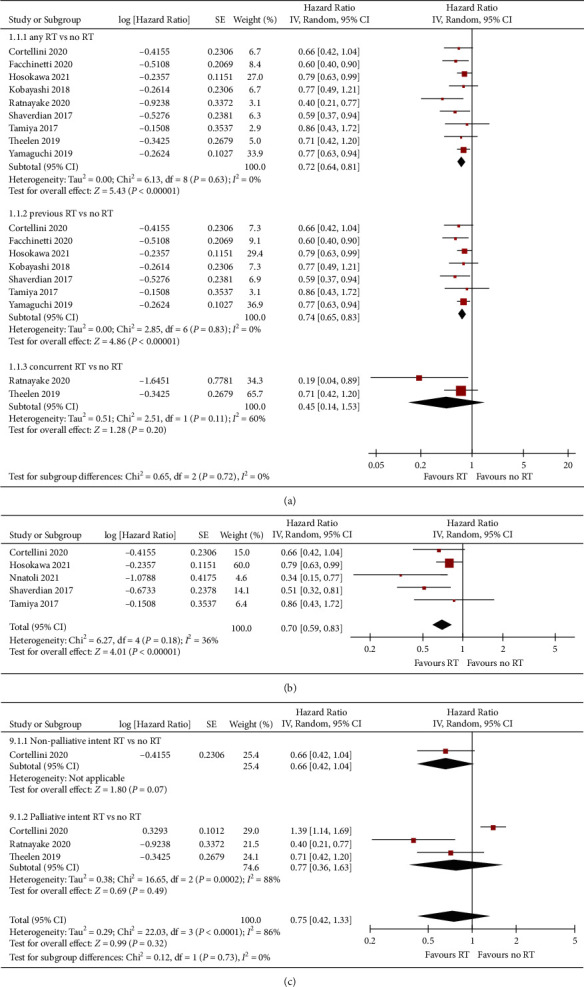
Meta-analysis of PFS in RT + ICIs versus ICIs in advanced NSCLC patients. (a) Meta-analysis of PFS between RT + ICIs and ICIs groups in the setting of different RT timing (prior versus concurrent). (b) Subgroup meta-analysis of RT + ICIs versus ICIs with regard to RT site (extracranial lesions). (c) Subgroup meta-analysis of PFS for RT + ICIs versus ICIs based on RT aims (non-palliative versus palliative RT intents).

The PFS outcomes of different groups were reported in 9 studies (Figure 3). For patients receiving RT + ICIs, the risk of disease progression was significantly lower than that of the ICIs group (HR = 0.72; 95% CI: 0.64, 0.81; *p* < 0.01). After dividing studies into two groups by RT timing, we found that patients in the prior RT group had a significantly better PFS (Figure 3(a)), while it was not significant in the concurrent RT group (Figure 3(a)). Further analysis of concurrent group based on RT types still failed to find a significant difference in PFS (Supplemental Figure 5). After including studies using RT to treat extracranial sites, these patients could still benefit from RT in terms of PFS with a statistical significance (HR = 0.70; 95% CI: 0.59, 0.83; *p* < 0.01) (Figure 3(b)). We also assessed whether the types of RT could influence the disease control benefits. As illustrated in Figure 3(c), although both non-palliative (HR = 0.66, *p* = 0.07) and palliative intent RT (HR = 0.77, *p* = 0.49) exhibited an obvious trend in reducing disease progression, it was not significant.

#### 3.4.4. OS

**Figure 4:**
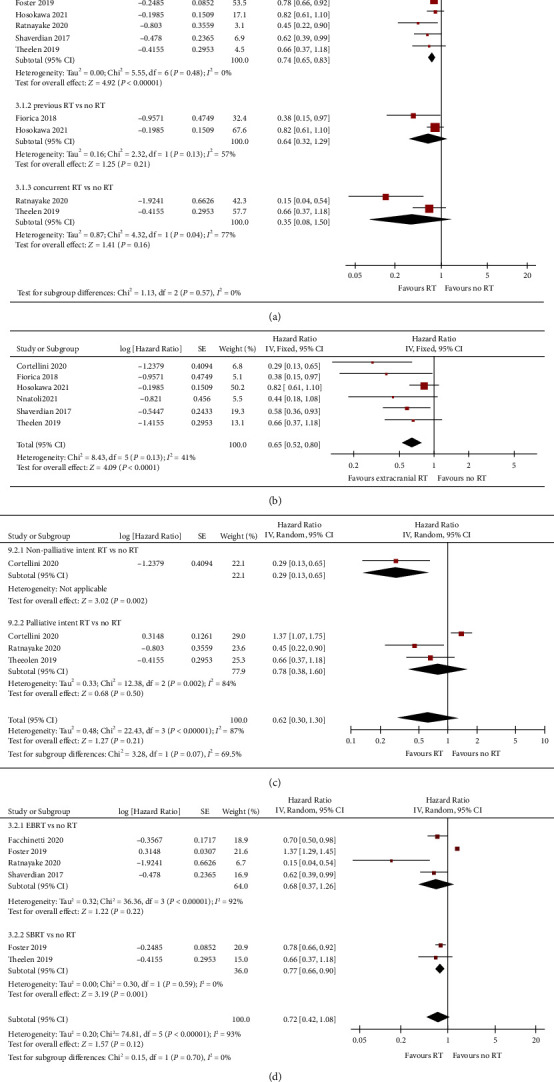
Meta-analysis of OS in RT + ICIs versus ICIs in advanced NSCLC patients. (a) Meta-analysis of OS between RT + ICIs and ICIs groups (prior versus concurrent). (b) Subgroup meta-analysis of RT + ICIs versus ICIs with regard to RT site (extracranial lesions). Subgroup meta-analysis of OS for RT + ICIs versus ICIs based on RT aims ((c) non-palliative versus palliative RT intents) and types ((d) CRT versus SBRT).

We extracted OS outcomes from 7 eligible studies (Figure 4(a)). Compared to ICIs alone group, the risk of death was significantly lower in the RT + ICIs group (HR = 0.74; 95% CI: 0.65, 0.83; *p* < 0.01). The meta-analysis showed that both the prior RT group (Figure 4(a)) and the concurrent RT group (Figure 4(a)) were associated with improved OS. However, this survival benefit was not significant after dividing studies into two groups by RT timing (prior RT: HR = 0.64; concurrent RT: HR = 0.35; *p* > 0.05 for all). The application to extracranial lesions resulted in a statistically significant improvement in OS compared to those in the ICIs alone group (HR = 0.65; 95% CI: 0.52, 0.80; *p* < 0.01) (Figure 4(b)). We performed another subgroup analysis to determine whether the types of RT could influence the death risk benefits. As illustrated in Figure 4(c), patients in the non-palliative RT group had a significantly better OS (HR = 0.29; 95% CI: 0.13, 0.65; *p* = 0.002), but not those in the palliative intent RT group (HR = 0.78, *p* = 0.50). Next, the impact of RT types on OS was also evaluated (Figure 4(d)). By dividing the studies into CRT and SBRT groups, the meta-analysis suggested that patients receiving SBRT had a significantly longer OS than those in the ICIs alone group (HR = 0.77; 95% CI: 0.66, 0.90; *p* = 0.001). However, it was not significant in the CRT group (HR = 0.68; 95% CI: 0.37, 1.26; *p* = 0.22). For this difference, the RT biological effective dose (BED) may be the reason. Compared to low BED group, high BED was associated with a better OS in patients treated with RT + ICIs (Supplemental Figure 6). In addition, a correlation analysis between BED and OS of the RT + ICIs was performed (Supplemental Figure 7), and the result suggested that there was an obvious correlation between BED and OS (number of pairs = 3; Pearson *r* = 0.86; squared *R* = 0.75; *p* = 0.34).

#### 3.4.5. Safety

**Figure 5:**
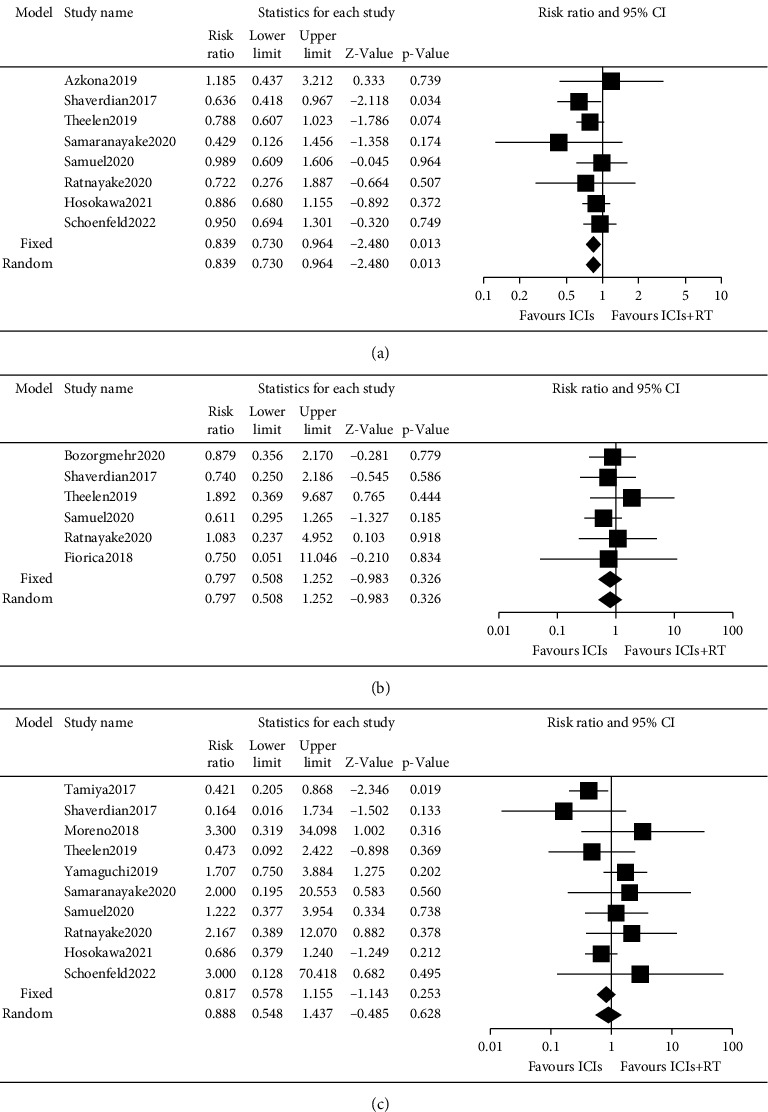
Meta-analysis of safety in advanced NSCLC patients who received ICIs versus ICIs + RT. (a) The overall risk ratio of any grade adverse events in patients treated with ICIs versus ICIs + RT. (b) The overall risk ratio of grade 3 or higher grade adverse events in patients treated with ICIs versus ICIs + RT. (c) The overall risk ratio of pneumonia in patients treated with ICIs versus ICIs + RT.

We summarized the safety data from included studies and performed meta-analyses. The overall risks of any adverse events, grade 3 or higher adverse events, and pneumonitis were analyzed (Figure 5). For ICIs versus RT + ICIs, the risk ratios of any adverse events (Figure 5(a)), grade 3 or higher adverse events (Figure 5(b)), and pneumonitis (Figure 5(c)) were 0.84 (95% CI: 0.73, 0.96; *p* = 0.01), 0.80 (95% CI: 0.51, 1.25; *p* = 0.33), and 0.89 (95% CI: 0.55, 1.44; *p* = 0.63), respectively.

#### 3.4.6. Sensitivity, Cumulative, and Publication Bias Assessments

As the analyses of ORR and DCR included most of the studies, sensitivity, cumulative, and publication bias assessments were performed using the data of ORR and DCR.

The sensitivity analyses of ORR were performed to examine whether the overall estimate could be significantly influenced by the included studies (Supplemental Figure 8). The results showed that the ORR was reliable when excluding each study at one time. The ORR cumulative analysis results showed that the ORR and its associated 95% CI were stable and in favor of the RT + ICIs treatment (Supplemental Figure 9). To detect potential publication bias, the funnel plot was applied and Begg's test and Egger's test were conducted by using the data of ORR (Supplemental Figure 10). The funnel plot of ORR showed that the risk of publication bias was low as the studies were located within the plot's range (Egger's test: *p* > 0.05; Begg's test: *p* > 0.05).

The results of the sensitivity analysis (Supplemental Figure 11) were reliable after removing each study included in the DCR analysis, and the DCR estimates were within their final confidence interval. The DCR cumulative analysis results (Supplemental Figure 12) showed that the DCR and its associated 95% CI were always in favor of RT + ICIs treatment. The funnel plot of DCR showed that the risk of publication bias was moderate as the studies were located within the plot's range (Supplemental Figure 13).

## 4. Discussion

The landscape of advanced NSCLC treatment has rapidly changed in recent years, especially after the emergence of ICIs. How to optimize the efficacy of this strategy is currently under investigation. In this study, the impact of RT on outcome and safety of advanced NSCLC patients treated with immunotherapy was assessed. The results found that the efficacy and survival outcomes were improved when adding RT to ICIs, with acceptable safety. Subgroup analyses suggested that patients who received non-palliative RT or SBRT had significant improvements in OS. Of note, PFS may serve as an indicator of OS in patients treated with RT + ICIs. These were in accordance with previously reported studies that using RT with ICIs may be associated with improved PFS and OS in well-selected patients [16, 31, 50].

Compared to ICIs alone, whether the combination of RT and ICIs has a superior effect on the efficacy and survival of advanced NSCLC patients is still under debate. Although evidence from a large number of studies showed improved efficacy after combination therapy of RT and ICIs, other results were also reported. Several studies [20, 21, 51] found that RT failed to significantly improve the PFS in the setting of immunotherapy. Of note, the study of Cortellini et al. suggested that previous palliative RT was significantly associated with shortened PFS and OS (*p* < 0.05 for all) in metastatic NSCLC patients with PD-L1 expression ≥50% [20], indicating that the RT types and PD-L1 level may affect the survival outcomes. After combining all the eligible studies, the overall effect was in favor of the combination group, suggesting that RT could improve the ORR and DCR in these patients. Also, this was further validated by the cumulative analysis and sensitivity analysis. Some studies [20–23, 38] did not support that the combination of RT and ICIs could have a better OS in treating patients with advanced NSCLC. In our analysis, the pooled estimate of OS was better in the RT + ICIs group in the setting of retrospective studies. Also, the OS of patients from the prospective studies was much longer for RT + ICIs than that of the ICIs group. These findings suggest that the administration of RT can improve the efficacy and survival in advanced NSCLC patients with ICIs treatment.

Although the above findings are promising, how to optimize the efficacy of RT + ICIs is unanswered. Based on the results of the PACIFIC study [14] and PEMBRO-RT [17], the RT timing and types may be associated with different treatment outcomes of immunotherapy. Therefore, several RCTs [52, 53] on the topic of RT timing and types in advanced NSCLC are ongoing and without conclusions. The definition of RT timing varied between studies, leading to various cutoff values of RT timing. Unlike other studies, the study of Kong et al. [22] was the main evidence supporting that prior RT was superior to concurrent RT in improving OS. They focused on the effect of thoracic radiotherapy on survival of stage IV NSCLC treated with and without immunotherapy. Patients who were treated with immunotherapy and thoracic radiotherapy concurrently had a worse OS (*n* = 177, median OS = 7.4 months, and *p* < 0.001), compared with those who had a thoracic radiotherapy history before immunotherapy ( *n* = 165, median OS = 12.2, months) [22]. All the above differences indicate that more studies that directly compare the various RT timing on the efficacy of ICIs are needed.

In our analyses, we found that previous RT was associated with significant improvement in PFS. The subgroup analysis of RT timing on OS suggested that neither prior RT nor concurrent RT was associated with significant improvements in OS, although there was a clear trend favoring RT + ICIs. The limited number of included studies may be responsible for these results. Another concern about this combination strategy is the dose and fraction of radiotherapy. Indeed, the SBRT was associated a significantly longer OS but not CRT. The BED may be responsible for this observation. Our correlation analysis suggested that BED was highly correlated with OS in patients treated with RT + ICIs. Moreover, this was in accordance with the findings of Foster et al. [39]. The impact of the types of RT on OS was also evaluated. Although patients may benefit from RT + ICIs treatment, patients who were treated with non-palliative RT could have a significantly lower risk of death but not palliative RT. Patients who were suitable for non-palliative RT may have a better disease burden or condition. Besides, they may receive higher dose of RT. These may result in the difference of OS.

Recently, a few meta-analyses [54–56] on the topic of ICI combination strategy have been published. The study of Mo et al. [54] included nine RCTs and compared the PFS and OS benefits from the combination therapy, such as immunotherapy plus chemotherapy, double immunotherapy agents, or immunotherapy plus targeted chemotherapy. The results of their study provided a clear suggestion that the risks of death and disease progression were significantly reduced when immunotherapy was combined with chemotherapy or other treatment options in NSCLC patients [54]. For immunotherapy plus RT, the study of Kim et al. [55] included studies of NSCLC patients with brain metastases, and the results showed that the combination group had a better intracranial local efficacy than that of ICI monotherapy. Another study by Voronova et al. [56] evaluated the impact of the schedule of RT on efficacy outcomes of brain metastases when combined with ICIs. After including 40 studies with 4359 patients, they found that RT concurrent with ICIs was associated with a better survival rate than the sequential combination group [56]. Though these findings are in accordance with our study, there are several differences. The meta-analysis by Voronova et al. [56] focused on comparing RT versus combination therapy, and we focused on ICIs versus RT + ICIs. First, the subjects in our study were advanced NSCLC patients treated with either ICIs or RT + ICIs, and the primary endpoints were efficacy and survival. Second, the impact of RT timing and disease condition on ORR, DCR, PFS, and OS in advanced NSCLC was evaluated in the subgroup analyses. Third, all relevant studies on the same topic were included, and subgroup analysis based on study design was performed, minimizing the risk of selection and inclusion bias. Nonetheless, this systematic review and meta-analysis evaluates the impact of RT sites, timing, and types on survival in advanced NSCLC patients treated with RT + ICIs versus ICIs alone.

The following limitations exist in the present meta-analysis. (1) Most of the included studies are retrospective analyses of ICIs versus RT + ICIs and may result in an increased risk of selection and reporting bias. Patients from most of the retrospective studies were likely to be different in disease conditions from those who did not receive radiation. Besides, the decisions of using radiotherapy as well as the timing and types of radiotherapy were associated with disease-related factors that would affect the outcome of these patients. These could introduce a high risk of selection bias. (2) Though the overall quality of the literature included in this meta-analysis is moderate, the risk of repeat reporting may exist among the included studies as few of the authors came from the same hospital, though they focused on different aspects of the disease and treatment. 11 of the studies were published on conferences and in abstract form, which may hamper the overall quality of our meta-analysis. (3) No language limitation is applied at the time of searching. However, the literature included in this study is only in English. It is not sure whether the results of the meta-analysis can be applied to all races/nations. (4) The differences between clinical characteristics, the definition of RT timing, plan of RT, RT dose and fraction, reporting of survival, and the inconsistency in the disease stage and degree of some underlying conditions may increase clinical heterogeneity between studies. For example, the number of metastasis lesions, different metastatic organs, and disease burden can impact the treatment effectiveness of ICIs. The varied doses and types of RT could result in a different tumor response. Therefore, a better analysis would be to obtain the source data from the previously published manuscripts to pool the data and analyze the data in a non-biased manner. (5) Not all the studies reported the primary endpoints, and thus a limited number of studies were included in the specific analysis, such as meta-analyses of ORR and DCR. This may underestimate or overestimate the actual effectiveness of RT + ICIs. Nevertheless, this systematic review and meta-analysis may answer some concerns and provide evidence for clinical practice.

In conclusion, the present meta-analysis suggests a combination of RT and ICIs to serve as a promising treatment strategy for improving the treatment efficacy of advanced NSCLC patients. However, its impact on survival needs to be further determined.

## Figures and Tables

**Table 1:** Baseline characteristics of included studies.

Author	Year	Study type	*N*	Region	Sex (male)	Age	Disease	RT details	ICI details	Treatment line	RT timing
A. Tamiya	2017	RS	201	Asia	135	68	Advanced NSCLC	Thoracic radiation	Nivolumab, 3 mg/kg, every two weeks	≥2nd line	Prior
N. Shaverdian	2017	PS	97	America	50	66	Advanced NSCLC	Extracranial radiation therapy or thoracic radiation	Pembrolizumab, 2 mg/kg or 10 mg/kg every 3 weeks or 10 mg/kg every 2 weeks	≥2nd line	Prior
A.M. Hegde	2018	RS	109	America	NR	NR	Advanced lung cancer	SBRT, EBRT	Nivolumab	≥1st line	Prior
D. Glick	2018	RS	271	America	NR	NR	Metastatic NSCLC	Palliative or curative RT	Nivolumab or pembrolizumab	≥1st line	Prior
F. Fiorica	2018	RS	35	Europe	30	69	Advanced lung cancer	Palliative RT: 3 to 12 Gy per fraction and a total dose of 8 to 36 Gy	Nivolumab, 3 mg/kg, every 2 weeks	≥2nd line	Prior
F.M.S. Kong	2018	RS	4639	America	NR	NR	Metastatic NSCLC	Thoracic radiation	NR	≥1st line	Multiple
Keigo Kobayashi	2018	RS	142	Asia	106	67	Advanced NSCLC	Palliative or curative RT	Nivolumab	≥2nd line	Prior
T. Sone	2018	RS	191	America	NR	NR	Advanced NSCLC	NR	NR	≥1st line	Prior
V. Moreno	2018	PS	53	America	35	64–67	Advanced NSCLC	Palliative RT	Cemiplimab	≥2nd line	Prior
C.C. Foster	2019	RS	5807	America	3031	NR	Metastatic NSCLC	EBRT: 30 Gy in a median of 10 fractions	Immunotherapy	≥1st line	Multiple
								SBRT: 22 Gy in a median of a single fraction			
E. Azkona	2019	RS	48	Europe	30	62	Advanced NSCLC	NR	NR	2nd line	Prior
J. W. Welsh	2019	RCT	36	America	NR	NR	Metastatic NSCLC	SBRT (50 Gy/4f or 70 Gy/10f) or traditional RT (45 Gy/15f)	Pembrolizumab, 200 mg, every 3 weeks for up to sixteen cycles	≥1st line	Multiple
K. A. D'Rummo	2019	RS	121	America	NR	NR	Metastatic NSCLC	NR	NR	≥1st line	Multiple
O. Yamaguchi	2019	RS	66	Asia	51	69	Advanced NSCLC	Chemoradiotherapy, palliative thoracic RT, palliative bone RT, and cranial RT	Nivolumab, 3 mg/kg every two weeks	2nd line	Prior
W. S. M. E. Theelen	2019	RCT	76	America	20	62	Advanced NSCLC	SBRT: 24 Gy/3f	Pembrolizumab, 200 mg every 3 weeks	≥1st line	Prior
A. Cortellini	2020	RS	1026	Multiple	NR	NR	Metastatic NSCLC	SBRT or palliative RT	Pembrolizumab	1st line	Prior
C. Samaranayake	2020	PS	46	Australia	18	63	Advanced NSCLC	53.3 ± 12.3 Gy radiation therapy	Nivolumab	≥2nd line	Prior
D. Chen	2020	RCT	97	America	NR	NR	Metastatic NSCLC	50 Gy/4f or 45 Gy/15f	Pembrolizumab, 100–200 mg, every 3 weeks, ≤16 cycles	≥1st line	Concurrent
E. Samuel	2020	RS	102	Australia	64	70	Metastatic NSCLC	Conventional hypofractionated, SRS, or both	Nivolumab or pembrolizumab	≥1st line	Concurrent
F. Bozorgmehr	2020	PS	101	Multiple	NR	NR	Advanced NSCLC	Palliative RT: 5 × 4 Gy to metastasis	Nivolumab, 240 mg, every 2 weeks	≥2nd line	Concurrent
F. Facchinetti	2020	RS	153	Europe	108	70	Advanced NSCLC	NR	Pembrolizumab	1st line	Prior
G. Ratnayake	2020	RS	85	Australia	44	65	Metastatic NSCLC	30 Gy (8–66 Gy), 20 Gy/5f, or 8 Gy/1f	Nivolumab, 3 mg/kg every 2 weeks	≥1st line	Multiple
S Hosokawa	2021	RS	531	Asia	420	68–69	Advanced NSCLC	Thoracic radiation	Anti-PD-1/L1	≥1st line	Prior
S. All	2021	RS	41	America	NR	NR	Metastatic NSCLC	Palliative or consolidative RT	Pembrolizumab	≥2nd line	Concurrent
G. Nnatoli	2021	RS	40	Europe	NR	NR	Metastatic NSCLC	Palliative radiation	Anti-PD1 or antiPD-L1	≥1st line	Concurrent
Schoenfeld, J. D	2022	RCT	26	Multiple	17 (65%)	65	Metastatic NSCLC	SBRT: 24 Gy/3f	Durvalumab; tremelimumab	≥3nd line	Concurrent

*N*, number; RT, radiotherapy; NR, not reported; NSCLC, non-small-cell lung cancer; ICIs, immune checkpoint inhibitors; SBRT, stereotactic body radiation therapy; EBRT, external beam radiation therapy; RS, retrospective studies; PS, prospective studies; RCTs, randomized controlled trials.

## Data Availability

All data generated or analyzed during this study are included in this article and its supplementary information files. The PRISMA checklist is attached as Supplemental Table 1. Other additional information can be accessed by contacting the corresponding author.

## Abbreviations

RT: Radiotherapy
CRT: Conventional RT
ICIs: Immune checkpoint inhibitors
NSCLC: Non-small-cell lung cancer
SBRT: Stereotactic ablative radiotherapy
PRISMA: Preferred Reporting Items for Systematic Reviews and Meta-Analyses
NOS: Newcastle-Ottawa Scale
OR: Odds ratio
RR: Risk ratio
MOD: Median of the difference
ORR: Objective response rate
DCR: Disease control rate
PFS: Progression-free survival
OS: Overall survival
TMB: Tumor mutation burden
NLR: Neutrophil-to-lymphocyte ratio
BMI: Body mass index
CI: Confidence interval
MD: Mean difference
SMD: Standardized mean difference
BED: Biological effective dose.

## Additional Points

Recent evidence suggests that the efficacy of immunotherapy is improved when adding radiotherapy to treat cancer. In this study, we assessed the impact of radiotherapy on the efficacy and survival of advanced NSCLC patients treated with immune checkpoint inhibitors. We found that patients in the combination group had better treatment response and survival benefits than those with immunotherapy alone. Subgroup analyses based on RT types and sites suggested that OS was longer in the combination group than that in the immunotherapy alone group. We did not observe a significant association between RT timing and the efficacy of combination therapy in advanced NSCLC.

## Supplementary Materials

Supplementary Materials Supplemental Table 1: the PRISMA checklist. Supplemental Table 2: the example of search strategy using Embase database. Supplemental Table 3: quality assessment of included studies by NOS. Supplemental Table 4: the methodological quality evaluation of included studies by the Cochrane Handbook methods for RCTs. Supplemental Figure 1: meta-analysis of ORR in advanced NSCLC patients treated with ICIs versus RT + ICIs regimen. (A) The OR of advanced NSCLC patients in ICIs versus RT + ICIs group in the setting of study designs. The combined OR is in favor of RT + ICIs group. (B) The OR of ICIs versus RT + ICIs based on the disease condition. The improvement of ORR is in favor of RT plus ICIs. (C) The OR of ICIs versus RT + ICIs according to RT timing. Supplemental Figure 2: the ORR meta-analyses in terms of RT types and immunotherapy treatment line. (A) The impact of RT types on OR of ORR for ICIs versus ICIs + RT. (B) The impact of treatment line on OR of ORR for ICIs versus ICIs + RT. Supplemental Figure 3: meta-analysis results of DCR in ICIs versus RT + ICIs groups for advanced NSCLC patients. (A) Meta-analysis of DCR between ICIs and RT + ICIs groups in the setting of different study designs. (B) Subgroup meta-analysis of ICIs versus RT + ICIs with regard to study design. (C) Subgroup meta-analysis of patients from ICIs versus RT + ICIs groups based on RT timing. Supplemental Figure 4: the DCR meta-analyses in terms of RT types and immunotherapy treatment line. (A) The impact of RT types on OR of DCR for ICIs versus ICIs + RT. (B) The impact of treatment line on OR of DCR for ICIs versus ICIs + RT. Supplemental Figure 5: meta-analysis of PFS based on RT types in the concurrent RT group. Supplemental Figure 6: meta-analysis of OS based on RT BED. Supplemental Figure 7: the correlation analysis between BED and OS from RT + ICIs group. Supplemental Figure 8: ORR sensitivity analysis. Supplemental Figure 9: cumulative analysis of ORR. Supplemental Figure 10: funnel plot of ORR. Supplemental Figure 11: sensitivity analysis of DCR. Supplemental Figure 12: cumulative analysis of DCR. Supplemental Figure 13: funnel plot of DCR.
