# Supplementary material for: Assessing Potential Factors Influencing the Efficacy of Immune Checkpoint Inhibitors with Radiation in Advanced Non-Small-Cell Lung Cancer Patients: A Systematic Review and Meta-Analysis
Source: J Oncol. 2023 Jan 13;2023:4477263. doi: 10.1155/2023/4477263 (PMC9859691; doi:10.1155/2023/4477263)

A

Group by RT types	Study name	Odds ratio	Lower limit	Upper limit	Z-Value	p-Value
ICI vs RT+ICI	Qin2017	0.050	0.002	1.067	-1.918	0.055
ICI vs RT+ICI	Tamiya2017	1.960	0.711	5.399	1.301	0.193
ICI vs RT+ICI	Kobayashi2018	0.315	0.128	0.774	-2.518	0.012
ICI vs RT+ICI	Moreno2018	0.333	0.095	1.173	-1.711	0.087
ICI vs RT+ICI	Theelen2019	0.982	0.189	5.108	-0.021	0.983
ICI vs RT+ICI	Welsh2019	1.167	0.393	3.467	0.277	0.781
ICI vs RT+ICI	Yamaguchi2019	0.410	0.179	0.936	-2.118	0.034
ICI vs RT+ICI	Bozorgmehr2020	3.855	1.031	14.415	2.005	0.045
ICI vs RT+ICI	Chen2020	0.380	0.105	1.381	-1.469	0.142
ICI vs RT+ICI	Cortellini2020	1.149	0.830	1.590	0.839	0.402
ICI vs RT+ICI	Samaranayake2020	0.481	0.119	1.945	-1.026	0.305
ICI vs RT+ICI	Samuel2020	0.543	0.319	0.923	-2.257	0.024
ICI vs RT+ICI	Hosokawa2020	0.739	0.479	1.140	-1.368	0.171
ICI vs RT+ICI		0.713	0.489	1.038	-1.766	0.077
ICI vs SBRT+ICI	Theelen2017	0.600	0.112	3.214	-0.597	0.551
ICI vs SBRT+ICI	Theelen2018	0.337	0.109	1.048	-1.879	0.060
ICI vs SBRT+ICI		0.404	0.158	1.034	-1.891	0.059

Odds ratio and 95% CI

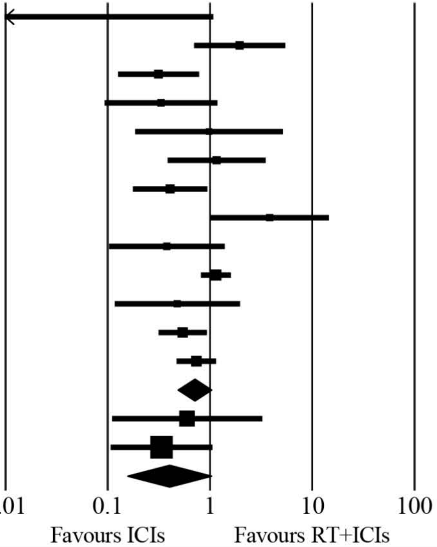

B

Model	Group by treatment line	Study name	Odds ratio	Lower limit	Upper limit	Z-Value	p-Value
	≥ 1st line	Samuel2020	0.543	0.319	0.923	-2.257	0.024
	≥ 1st line	Hosokawa2020	0.739	0.479	1.140	-1.368	0.171
Fixed	≥ 1st line		0.653	0.467	0.914	-2.487	0.013
Random	≥ 1st line		0.653	0.467	0.914	-2.487	0.013
	≥ 2nd line	Tamiya2017	1.960	0.711	5.399	1.301	0.193
	≥ 2nd line	Kobayashi2018	0.315	0.128	0.774	-2.518	0.012
	≥ 2nd line	Moreno2018	0.333	0.095	1.173	-1.711	0.087
	≥ 2nd line	Theelen2019	0.982	0.189	5.108	-0.021	0.983
	≥ 2nd line	Yamaguchi2019	0.410	0.179	0.936	-2.118	0.034
	≥ 2nd line	Bozorgmehr2020	3.855	1.031	14.415	2.005	0.045
	≥ 2nd line	Samaranayake2020	0.481	0.119	1.945	-1.026	0.305
Fixed	≥ 2nd line		0.662	0.436	1.003	-1.944	0.052
Random	≥ 2nd line		0.731	0.355	1.504	-0.851	0.395
Fixed	Overall		0.657	0.506	0.853	-3.156	0.002
Random	Overall		0.667	0.492	0.904	-2.614	0.009

Odds ratio and 95% CI

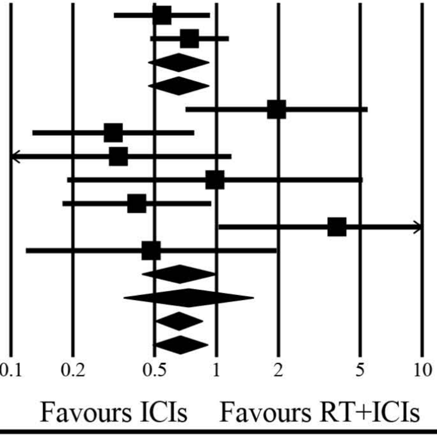

Supplement: Supplementary Materials — Supplemental Table 1: the PRISMA checklist. Supplemental Table 2: the example of search strategy using Embase database. Supplemental Table 3: quality assessment of included studies by NOS. Supplemental Table 4: the methodological quality evaluation of included studies by the Cochrane Handbook methods for RCTs. Supplemental Figure 1: meta-analysis of ORR in advanced NSCLC patients treated with ICIs versus RT + ICIs regimen. (A) The OR of advanced NSCLC patients in ICIs versus RT + ICIs group in the setting of study designs. The combined OR is in favor of RT + ICIs group. (B) The OR of ICIs versus RT + ICIs based on the disease condition. The improvement of ORR is in favor of RT plus ICIs. (C) The OR of ICIs versus RT + ICIs according to RT timing. Supplemental Figure 2: the ORR meta-analyses in terms of RT types and immunotherapy treatment line. (A) The impact of RT types on OR of ORR for ICIs versus ICIs + RT. (B) The impact of treatment line on OR of ORR for ICIs versus ICIs + RT. Supplemental Figure 3: meta-analysis results of DCR in ICIs versus RT + ICIs groups for advanced NSCLC patients. (A) Meta-analysis of DCR between ICIs and RT + ICIs groups in the setting of different study designs. (B) Subgroup meta-analysis of ICIs versus RT + ICIs with regard to study design. (C) Subgroup meta-analysis of patients from ICIs versus RT + ICIs groups based on RT timing. Supplemental Figure 4: the DCR meta-analyses in terms of RT types and immunotherapy treatment line. (A) The impact of RT types on OR of DCR for ICIs versus ICIs + RT. (B) The impact of treatment line on OR of DCR for ICIs versus ICIs + RT. Supplemental Figure 5: meta-analysis of PFS based on RT types in the concurrent RT group. Supplemental Figure 6: meta-analysis of OS based on RT BED. Supplemental Figure 7: the correlation analysis between BED and OS from RT + ICIs group. Supplemental Figure 8: ORR sensitivity analysis. Supplemental Figure 9: cumulative analysis of ORR. Supplemental Figure 10: funnel plot [file 4477263.f1.zip › Supplemental Figure 2 ORR updated 1 RT types and treatment line.pdf]
