# Supplementary material for: Assessing Potential Factors Influencing the Efficacy of Immune Checkpoint Inhibitors with Radiation in Advanced Non-Small-Cell Lung Cancer Patients: A Systematic Review and Meta-Analysis
Source: J Oncol. 2023 Jan 13;2023:4477263. doi: 10.1155/2023/4477263 (PMC9859691; doi:10.1155/2023/4477263)
Supplement: Supplementary Materials — Supplemental Table 1: the PRISMA checklist. Supplemental Table 2: the example of search strategy using Embase database. Supplemental Table 3: quality assessment of included studies by NOS. Supplemental Table 4: the methodological quality evaluation of included studies by the Cochrane Handbook methods for RCTs. Supplemental Figure 1: meta-analysis of ORR in advanced NSCLC patients treated with ICIs versus RT + ICIs regimen. (A) The OR of advanced NSCLC patients in ICIs versus RT + ICIs group in the setting of study designs. The combined OR is in favor of RT + ICIs group. (B) The OR of ICIs versus RT + ICIs based on the disease condition. The improvement of ORR is in favor of RT plus ICIs. (C) The OR of ICIs versus RT + ICIs according to RT timing. Supplemental Figure 2: the ORR meta-analyses in terms of RT types and immunotherapy treatment line. (A) The impact of RT types on OR of ORR for ICIs versus ICIs + RT. (B) The impact of treatment line on OR of ORR for ICIs versus ICIs + RT. Supplemental Figure 3: meta-analysis results of DCR in ICIs versus RT + ICIs groups for advanced NSCLC patients. (A) Meta-analysis of DCR between ICIs and RT + ICIs groups in the setting of different study designs. (B) Subgroup meta-analysis of ICIs versus RT + ICIs with regard to study design. (C) Subgroup meta-analysis of patients from ICIs versus RT + ICIs groups based on RT timing. Supplemental Figure 4: the DCR meta-analyses in terms of RT types and immunotherapy treatment line. (A) The impact of RT types on OR of DCR for ICIs versus ICIs + RT. (B) The impact of treatment line on OR of DCR for ICIs versus ICIs + RT. Supplemental Figure 5: meta-analysis of PFS based on RT types in the concurrent RT group. Supplemental Figure 6: meta-analysis of OS based on RT BED. Supplemental Figure 7: the correlation analysis between BED and OS from RT + ICIs group. Supplemental Figure 8: ORR sensitivity analysis. Supplemental Figure 9: cumulative analysis of ORR. Supplemental Figure 10: funnel plot [file 4477263.f1.zip › Supplemental Table 2.docx]

Supplemental Table 2 Example of Preliminary Embase search strategy

Embase

Session Results

.......................................................

| No. | Query Results |  | Date |
| --- | --- | --- | --- |
| #5. | #3 AND (2019: py OR 2020:py) AND ('clinical article'/de OR 'clinical trial'/de OR 'cohort analysis'/de OR 'controlled clinical trial'/de OR 'controlled study'/de OR 'human'/de OR 'major clinical study'/de OR 'multicenter study'/de OR 'phase 2 clinical trial'/de OR 'phase 2 clinical trial topic'/de OR 'phase 3 clinical trial'/de OR 'phase 3 clinical trial topic'/de OR 'prospective study'/de OR 'randomized controlled trial'/de OR 'randomized controlled trial topic'/de OR 'retrospective study'/de) AND ('Article'/it OR 'Article in Press'/it OR 'Conference Abstract'/it OR 'Conference Paper'/it) AND ('advanced cancer'/dm OR 'brain metastasis'/dm OR 'glioblastoma'/dm OR 'head and neck squamous cell carcinoma'/dm OR 'liver cell carcinoma'/dm OR 'lung adenocarcinoma'/dm OR 'lung cancer'/dm OR 'lung metastasis'/dm OR 'melanoma'/dm OR 'metastatic melanoma'/dm OR 'non small cell lung cancer'/dm OR 'small cell lung cancer'/dm OR 'squamous cell carcinoma'/dm) |  | 1 Oct 2020 |
| #4. | #3 AND (2019:py OR 2020:py) AND ('clinical article'/de OR 'clinical trial'/de OR 'cohort analysis'/de OR 'controlled clinical trial'/de OR 'controlled study'/de OR 'human'/de OR 'major clinical study'/de OR 'multicenter study'/de OR 'phase 2 clinical trial'/de OR 'phase 2 clinical trial topic'/de OR 'phase 3 clinical trial'/de OR 'phase 3 clinical trial topic'/de OR 'prospective study'/de OR 'randomized controlled trial'/de OR 'randomized controlled trial topic'/de OR 'retrospective study'/de) AND ('Article'/it OR 'Article in Press'/it OR 'Conference Abstract'/it OR 'Conference Paper'/it) |  | 1 Oct 2020 |
| #3. | #1 AND #2 |  | 1 Oct 2020 |
| #2. | ('malignant neoplasm'/exp OR 'neoplasm'/exp OR 'carcinoma'/exp OR 'glioma'/exp OR 'melanoma'/exp) AND ('radiotherapy'/exp OR 'radiation'/exp OR 'stereotactic body radiation therapy'/exp OR sabr) |  | 1 Oct 2020 |
| #1. | ('malignant neoplasm'/exp OR 'neoplasm'/exp OR 'carcinoma'/exp OR 'glioma'/exp OR 'melanoma'/exp) AND (a AND 'immune checkpoint inhibitor'/exp OR 'pd-1 inhibitor' OR 'ctla-4 inhibitor' OR 'nivolumab'/exp OR 'pembrolizumab'/exp OR 'atezolizumab'/exp OR 'ipilimumab'/exp) |  | 1 Oct 2020 |
