# Supplementary material for: Assessing Potential Factors Influencing the Efficacy of Immune Checkpoint Inhibitors with Radiation in Advanced Non-Small-Cell Lung Cancer Patients: A Systematic Review and Meta-Analysis
Source: J Oncol. 2023 Jan 13;2023:4477263. doi: 10.1155/2023/4477263 (PMC9859691; doi:10.1155/2023/4477263)
Supplement: Supplementary Materials — Supplemental Table 1: the PRISMA checklist. Supplemental Table 2: the example of search strategy using Embase database. Supplemental Table 3: quality assessment of included studies by NOS. Supplemental Table 4: the methodological quality evaluation of included studies by the Cochrane Handbook methods for RCTs. Supplemental Figure 1: meta-analysis of ORR in advanced NSCLC patients treated with ICIs versus RT + ICIs regimen. (A) The OR of advanced NSCLC patients in ICIs versus RT + ICIs group in the setting of study designs. The combined OR is in favor of RT + ICIs group. (B) The OR of ICIs versus RT + ICIs based on the disease condition. The improvement of ORR is in favor of RT plus ICIs. (C) The OR of ICIs versus RT + ICIs according to RT timing. Supplemental Figure 2: the ORR meta-analyses in terms of RT types and immunotherapy treatment line. (A) The impact of RT types on OR of ORR for ICIs versus ICIs + RT. (B) The impact of treatment line on OR of ORR for ICIs versus ICIs + RT. Supplemental Figure 3: meta-analysis results of DCR in ICIs versus RT + ICIs groups for advanced NSCLC patients. (A) Meta-analysis of DCR between ICIs and RT + ICIs groups in the setting of different study designs. (B) Subgroup meta-analysis of ICIs versus RT + ICIs with regard to study design. (C) Subgroup meta-analysis of patients from ICIs versus RT + ICIs groups based on RT timing. Supplemental Figure 4: the DCR meta-analyses in terms of RT types and immunotherapy treatment line. (A) The impact of RT types on OR of DCR for ICIs versus ICIs + RT. (B) The impact of treatment line on OR of DCR for ICIs versus ICIs + RT. Supplemental Figure 5: meta-analysis of PFS based on RT types in the concurrent RT group. Supplemental Figure 6: meta-analysis of OS based on RT BED. Supplemental Figure 7: the correlation analysis between BED and OS from RT + ICIs group. Supplemental Figure 8: ORR sensitivity analysis. Supplemental Figure 9: cumulative analysis of ORR. Supplemental Figure 10: funnel plot [file 4477263.f1.zip › Supplemental Table 3 NOS (1).docx]

Supplemental Table 3 Quality assessment of included studies by NOS

| Author | Year | Selection | | | | Comparability | Outcome | | | Score |
| --- | --- | --- | --- | --- | --- | --- | --- | --- | --- | --- |
|  |  | A | B | C | D | E | F | G | H |  |
| A. Cortellini | 2020 | ☆ | ☆ | ☆ | ☆ | ☆☆ | ☆ | ☆ |  | 8 |
| A. Hegde | 2018 | ☆ | ☆ | ☆ |  | ☆☆ | ☆ | ☆ |  | 7 |
| A. Tamiya | 2017 | ☆ | ☆ | ☆ | ☆ | ☆☆ | ☆ | ☆ | ☆ | 9 |
| C. Samaranayake | 2020 | ☆ | ☆ | ☆ | ☆ | ☆☆ | ☆ | ☆ |  | 8 |
| C.C. Foster | 2019 | ☆ | ☆ | ☆ | ☆ | ☆☆ | ☆ | ☆ |  | 8 |
| D. Glick | 2018 | ☆ | ☆ | ☆ | ☆ | ☆☆ | ☆ | ☆ |  | 8 |
| E. Azkona | 2019 | ☆ | ☆ | ☆ | ☆ | ☆☆ | ☆ | ☆ |  | 8 |
| E. Samuel | 2020 | ☆ | ☆ | ☆ | ☆ | ☆☆ | ☆ | ☆ |  | 8 |
| F. Bozorgmehr | 2020 | ☆ | ☆ | ☆ |  | ☆☆ | ☆ | ☆ |  | 7 |
| F. Facchinetti | 2020 | ☆ | ☆ | ☆ | ☆ | ☆☆ | ☆ | ☆ | ☆ | 9 |
| F. Fiorica | 2018 | ☆ | ☆ | ☆ | ☆ | ☆☆ | ☆ | ☆ | ☆ | 9 |
| F.M.S. Kong | 2018 | ☆ | ☆ | ☆ |  | ☆☆ | ☆ | ☆ |  | 7 |
| G. Ratnayake | 2020 | ☆ | ☆ | ☆ | ☆ | ☆☆ | ☆ | ☆ |  | 8 |
| K.A. D'Rummo | 2019 | ☆ | ☆ | ☆ | ☆ | ☆☆ | ☆ | ☆ |  | 8 |
| Keigo Kobayashi | 2018 | ☆ | ☆ | ☆ | ☆ | ☆☆ | ☆ | ☆ |  | 8 |
| N. Shaverdian | 2017 | ☆ | ☆ | ☆ |  | ☆☆ | ☆ | ☆ |  | 7 |
| O. Yamaguchi | 2019 | ☆ | ☆ | ☆ | ☆ | ☆☆ | ☆ | ☆ | ☆ | 9 |
| S Hosokawa | 2021 | ☆ | ☆ | ☆ | ☆ | ☆☆ | ☆ | ☆ |  | 8 |
| T. Sone | 2018 | ☆ | ☆ | ☆ |  | ☆☆ | ☆ | ☆ |  | 7 |
| V. Moreno | 2018 | ☆ | ☆ | ☆ |  | ☆☆ | ☆ | ☆ |  | 7 |
| S. All | 2021 | ☆ | ☆ | ☆ |  | ☆☆ | ☆ | ☆ |  | 7 |
| G. Nnatoli | 2021 | ☆ | ☆ | ☆ |  | ☆☆ | ☆ | ☆ |  | 7 |

Note: 1 ”Selection” part includes A: representativeness of cases, B: selection of controls, C: exposure ascertainment, and D: no death when investigation begin.2 ”Comparability” part includes E: comparable on confounders.3 ”Outcome” part includes F: outcome assessment, G: adequate follow-up, and H: loss to follow-up rate.4 The total score is equal to the total number of stars.
