# Supplementary figures and images for: Assessing Potential Factors Influencing the Efficacy of Immune Checkpoint Inhibitors with Radiation in Advanced Non-Small-Cell Lung Cancer Patients: A Systematic Review and Meta-Analysis
Source: J Oncol. 2023 Jan 13;2023:4477263. doi: 10.1155/2023/4477263 (PMC9859691; doi:10.1155/2023/4477263)

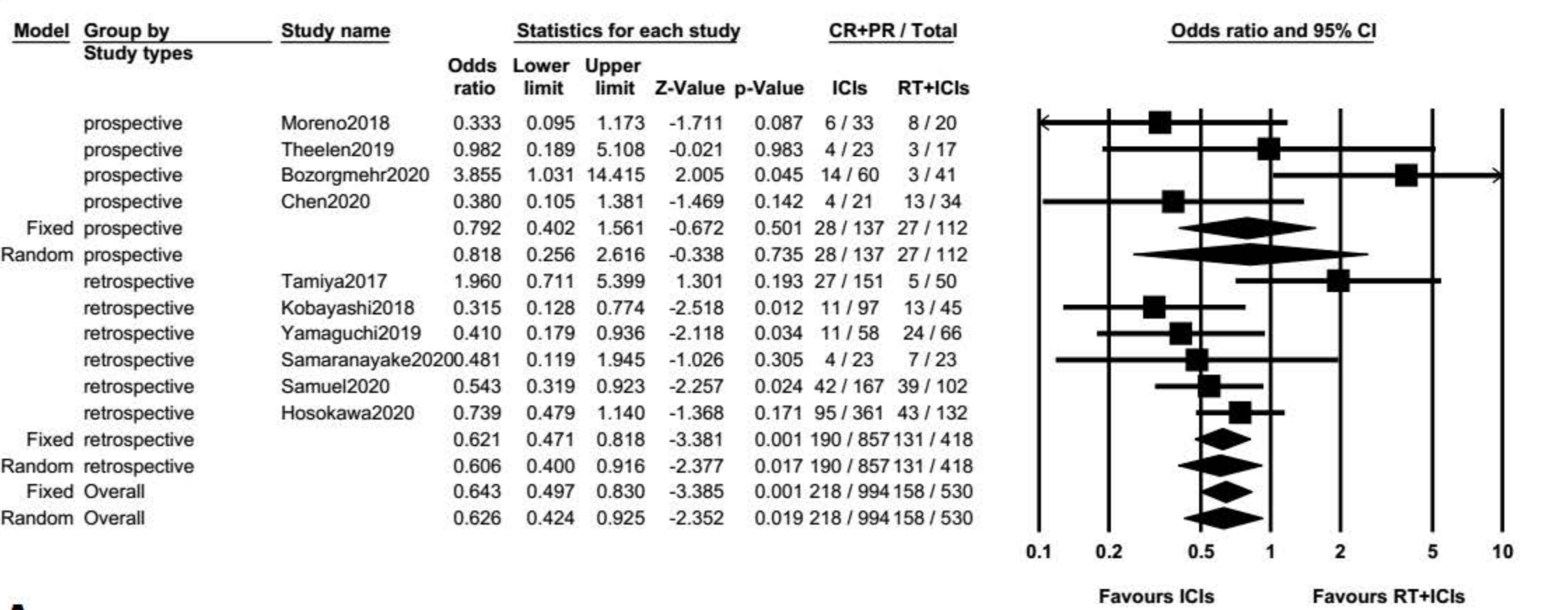

A

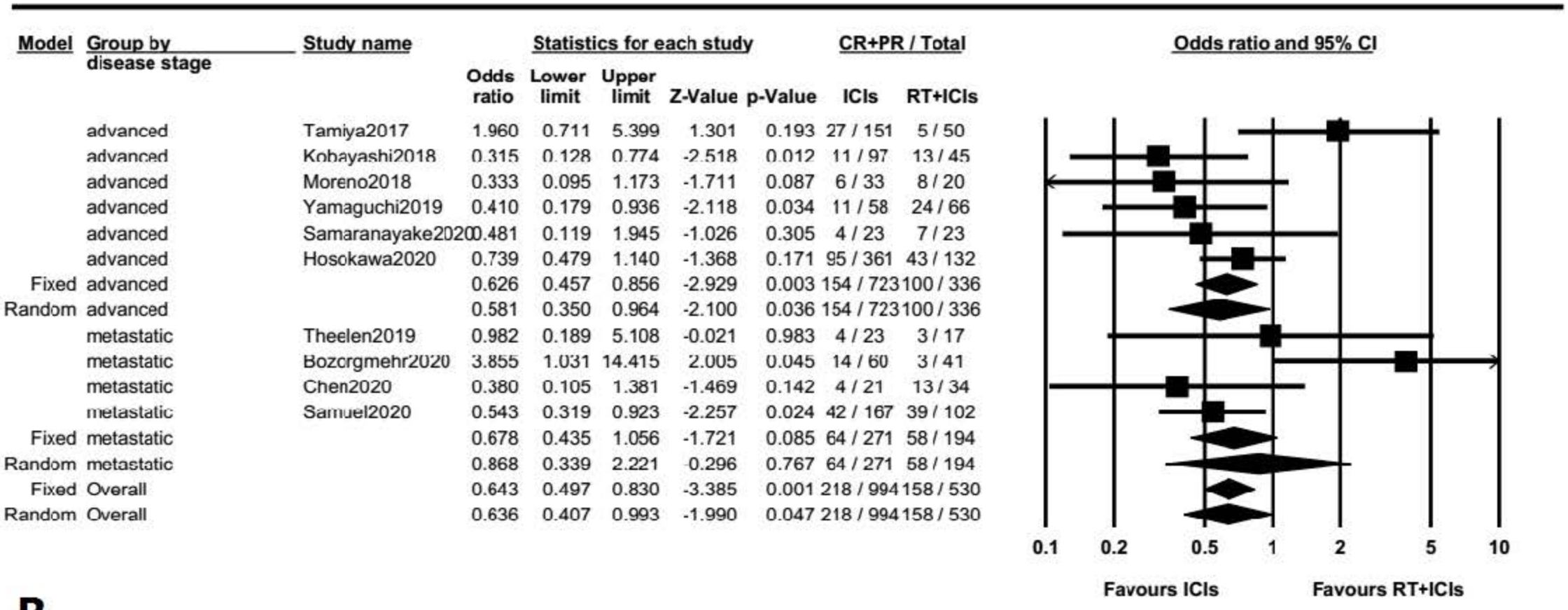

B

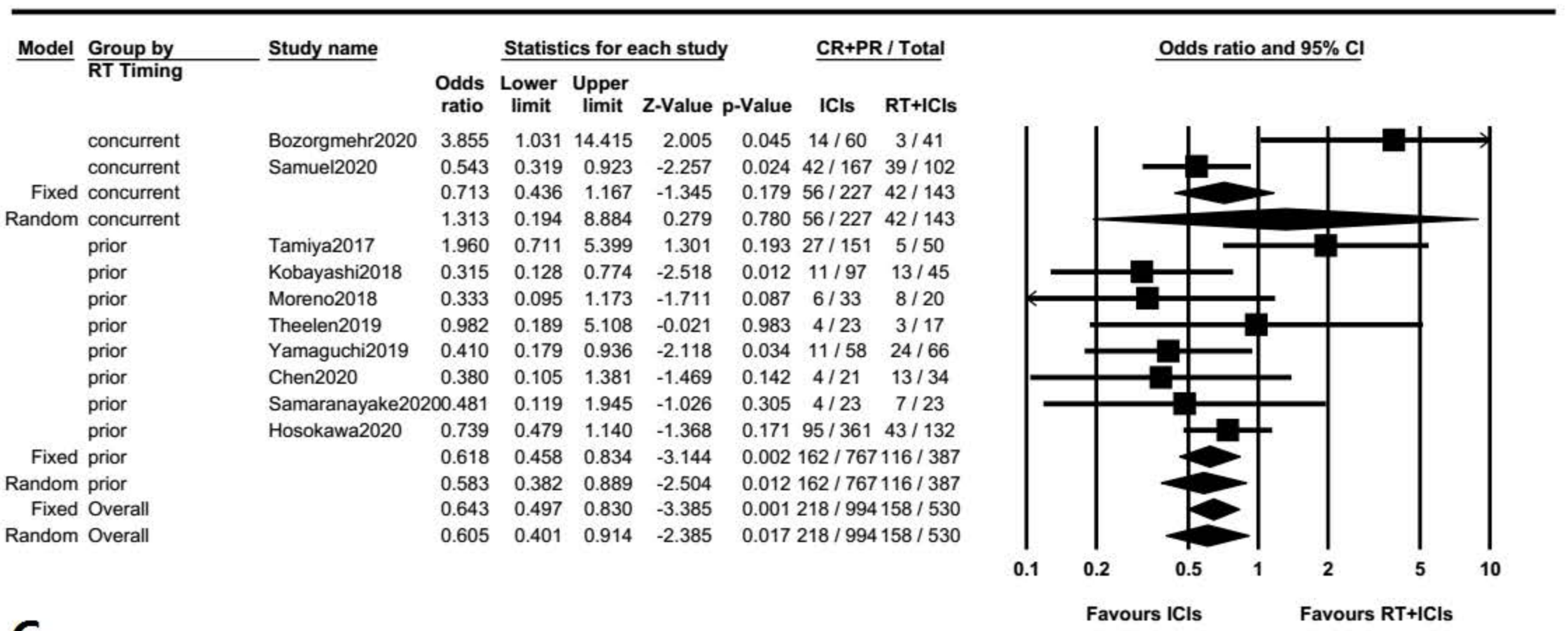

C

Supplement: Supplementary Materials — Supplemental Table 1: the PRISMA checklist. Supplemental Table 2: the example of search strategy using Embase database. Supplemental Table 3: quality assessment of included studies by NOS. Supplemental Table 4: the methodological quality evaluation of included studies by the Cochrane Handbook methods for RCTs. Supplemental Figure 1: meta-analysis of ORR in advanced NSCLC patients treated with ICIs versus RT + ICIs regimen. (A) The OR of advanced NSCLC patients in ICIs versus RT + ICIs group in the setting of study designs. The combined OR is in favor of RT + ICIs group. (B) The OR of ICIs versus RT + ICIs based on the disease condition. The improvement of ORR is in favor of RT plus ICIs. (C) The OR of ICIs versus RT + ICIs according to RT timing. Supplemental Figure 2: the ORR meta-analyses in terms of RT types and immunotherapy treatment line. (A) The impact of RT types on OR of ORR for ICIs versus ICIs + RT. (B) The impact of treatment line on OR of ORR for ICIs versus ICIs + RT. Supplemental Figure 3: meta-analysis results of DCR in ICIs versus RT + ICIs groups for advanced NSCLC patients. (A) Meta-analysis of DCR between ICIs and RT + ICIs groups in the setting of different study designs. (B) Subgroup meta-analysis of ICIs versus RT + ICIs with regard to study design. (C) Subgroup meta-analysis of patients from ICIs versus RT + ICIs groups based on RT timing. Supplemental Figure 4: the DCR meta-analyses in terms of RT types and immunotherapy treatment line. (A) The impact of RT types on OR of DCR for ICIs versus ICIs + RT. (B) The impact of treatment line on OR of DCR for ICIs versus ICIs + RT. Supplemental Figure 5: meta-analysis of PFS based on RT types in the concurrent RT group. Supplemental Figure 6: meta-analysis of OS based on RT BED. Supplemental Figure 7: the correlation analysis between BED and OS from RT + ICIs group. Supplemental Figure 8: ORR sensitivity analysis. Supplemental Figure 9: cumulative analysis of ORR. Supplemental Figure 10: funnel plot [file 4477263.f1.zip › Supplemental Figure 1 ORR.pdf]

**Funnel Plot of Standard Error by Log odds ratio**

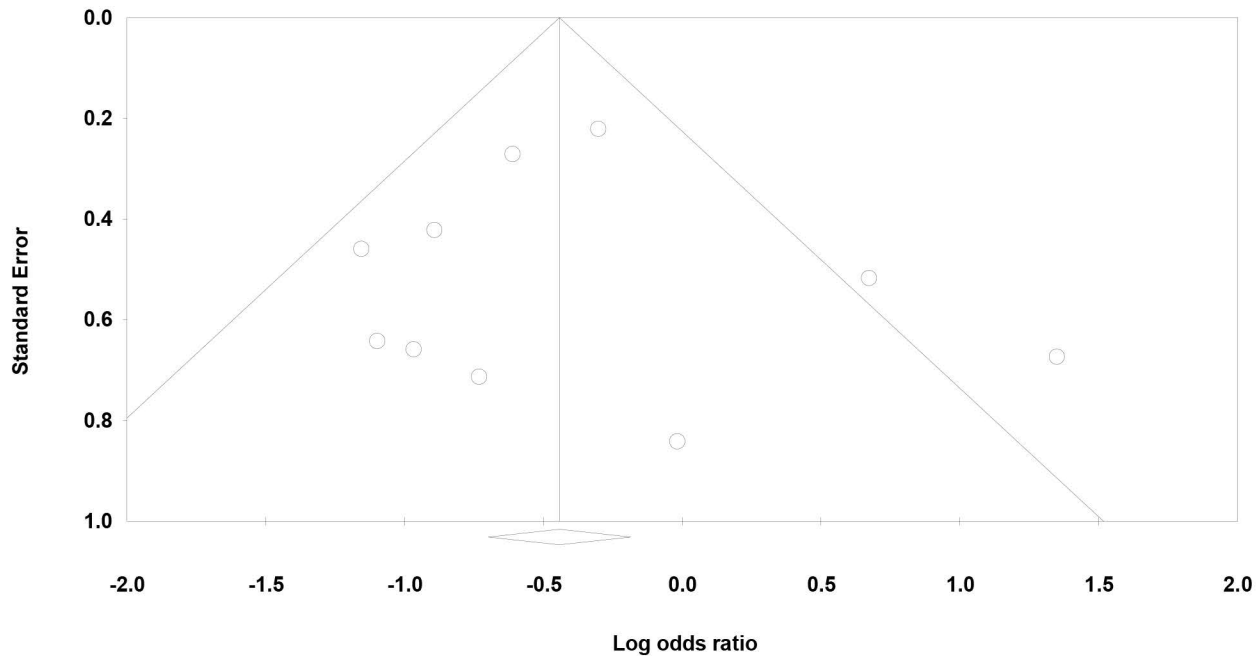

Supplement: Supplementary Materials — Supplemental Table 1: the PRISMA checklist. Supplemental Table 2: the example of search strategy using Embase database. Supplemental Table 3: quality assessment of included studies by NOS. Supplemental Table 4: the methodological quality evaluation of included studies by the Cochrane Handbook methods for RCTs. Supplemental Figure 1: meta-analysis of ORR in advanced NSCLC patients treated with ICIs versus RT + ICIs regimen. (A) The OR of advanced NSCLC patients in ICIs versus RT + ICIs group in the setting of study designs. The combined OR is in favor of RT + ICIs group. (B) The OR of ICIs versus RT + ICIs based on the disease condition. The improvement of ORR is in favor of RT plus ICIs. (C) The OR of ICIs versus RT + ICIs according to RT timing. Supplemental Figure 2: the ORR meta-analyses in terms of RT types and immunotherapy treatment line. (A) The impact of RT types on OR of ORR for ICIs versus ICIs + RT. (B) The impact of treatment line on OR of ORR for ICIs versus ICIs + RT. Supplemental Figure 3: meta-analysis results of DCR in ICIs versus RT + ICIs groups for advanced NSCLC patients. (A) Meta-analysis of DCR between ICIs and RT + ICIs groups in the setting of different study designs. (B) Subgroup meta-analysis of ICIs versus RT + ICIs with regard to study design. (C) Subgroup meta-analysis of patients from ICIs versus RT + ICIs groups based on RT timing. Supplemental Figure 4: the DCR meta-analyses in terms of RT types and immunotherapy treatment line. (A) The impact of RT types on OR of DCR for ICIs versus ICIs + RT. (B) The impact of treatment line on OR of DCR for ICIs versus ICIs + RT. Supplemental Figure 5: meta-analysis of PFS based on RT types in the concurrent RT group. Supplemental Figure 6: meta-analysis of OS based on RT BED. Supplemental Figure 7: the correlation analysis between BED and OS from RT + ICIs group. Supplemental Figure 8: ORR sensitivity analysis. Supplemental Figure 9: cumulative analysis of ORR. Supplemental Figure 10: funnel plot [file 4477263.f1.zip › Supplemental Figure 10 ORR funnel polt.pdf]

**Funnel Plot of Standard Error by Log odds ratio**

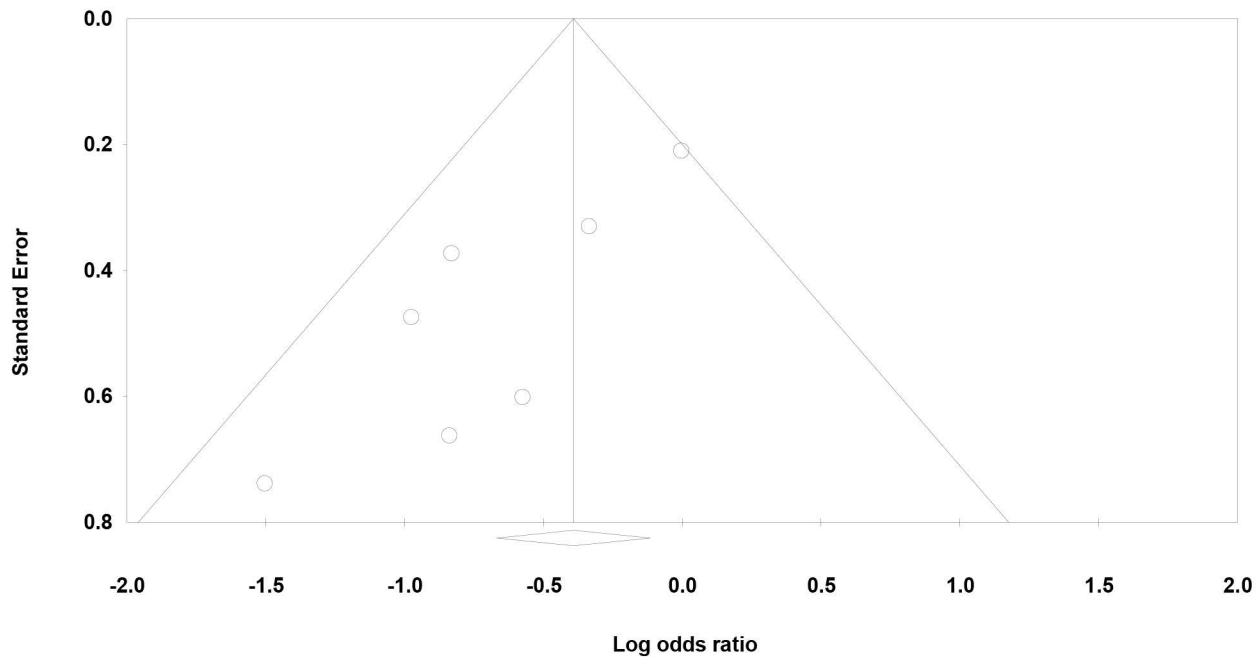

Supplement: Supplementary Materials — Supplemental Table 1: the PRISMA checklist. Supplemental Table 2: the example of search strategy using Embase database. Supplemental Table 3: quality assessment of included studies by NOS. Supplemental Table 4: the methodological quality evaluation of included studies by the Cochrane Handbook methods for RCTs. Supplemental Figure 1: meta-analysis of ORR in advanced NSCLC patients treated with ICIs versus RT + ICIs regimen. (A) The OR of advanced NSCLC patients in ICIs versus RT + ICIs group in the setting of study designs. The combined OR is in favor of RT + ICIs group. (B) The OR of ICIs versus RT + ICIs based on the disease condition. The improvement of ORR is in favor of RT plus ICIs. (C) The OR of ICIs versus RT + ICIs according to RT timing. Supplemental Figure 2: the ORR meta-analyses in terms of RT types and immunotherapy treatment line. (A) The impact of RT types on OR of ORR for ICIs versus ICIs + RT. (B) The impact of treatment line on OR of ORR for ICIs versus ICIs + RT. Supplemental Figure 3: meta-analysis results of DCR in ICIs versus RT + ICIs groups for advanced NSCLC patients. (A) Meta-analysis of DCR between ICIs and RT + ICIs groups in the setting of different study designs. (B) Subgroup meta-analysis of ICIs versus RT + ICIs with regard to study design. (C) Subgroup meta-analysis of patients from ICIs versus RT + ICIs groups based on RT timing. Supplemental Figure 4: the DCR meta-analyses in terms of RT types and immunotherapy treatment line. (A) The impact of RT types on OR of DCR for ICIs versus ICIs + RT. (B) The impact of treatment line on OR of DCR for ICIs versus ICIs + RT. Supplemental Figure 5: meta-analysis of PFS based on RT types in the concurrent RT group. Supplemental Figure 6: meta-analysis of OS based on RT BED. Supplemental Figure 7: the correlation analysis between BED and OS from RT + ICIs group. Supplemental Figure 8: ORR sensitivity analysis. Supplemental Figure 9: cumulative analysis of ORR. Supplemental Figure 10: funnel plot [file 4477263.f1.zip › Supplemental Figure 13 DCR funnel polt.pdf]

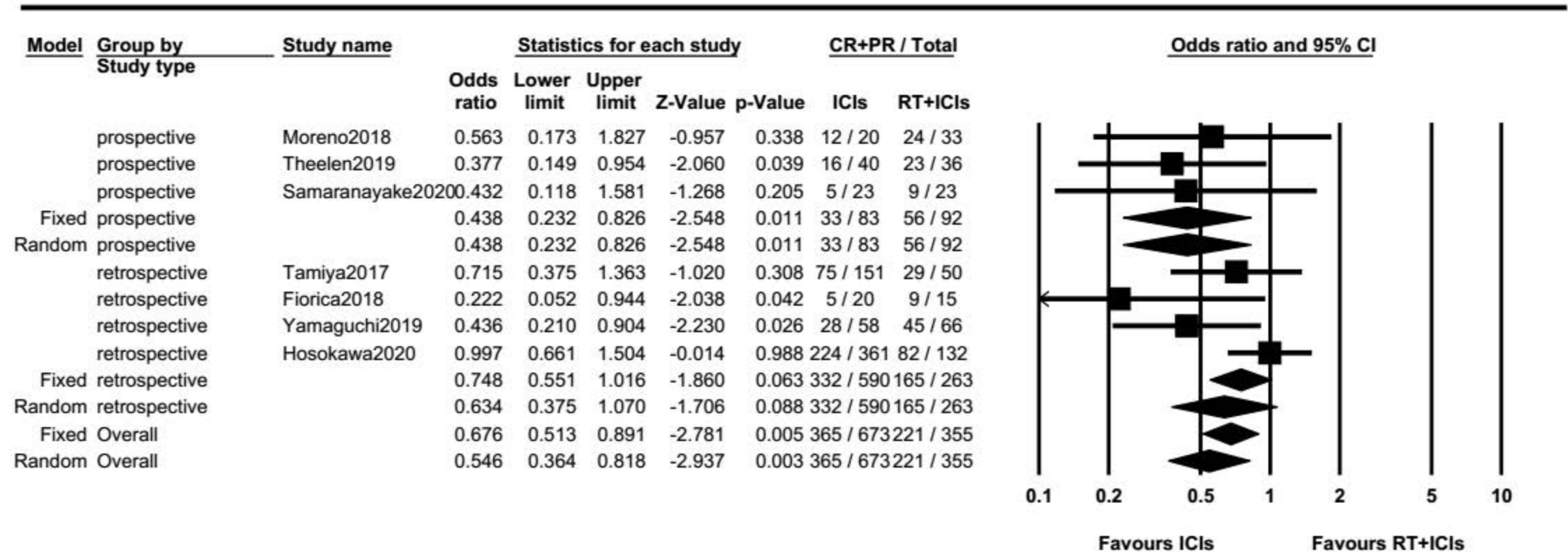

A

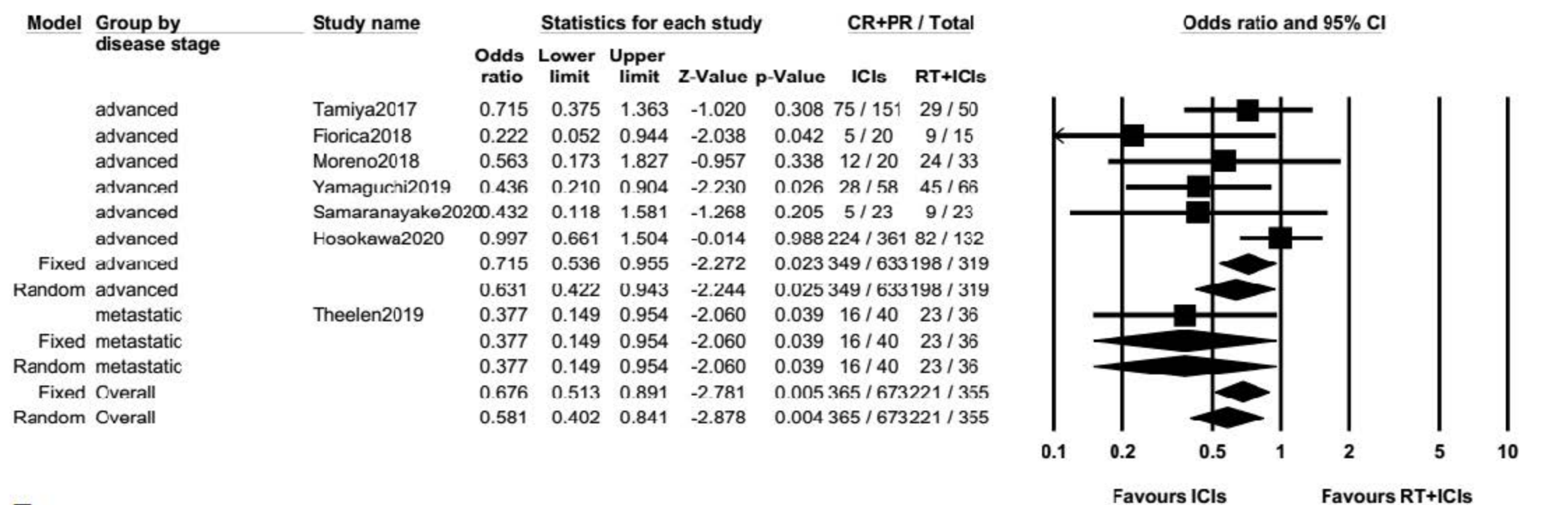

B

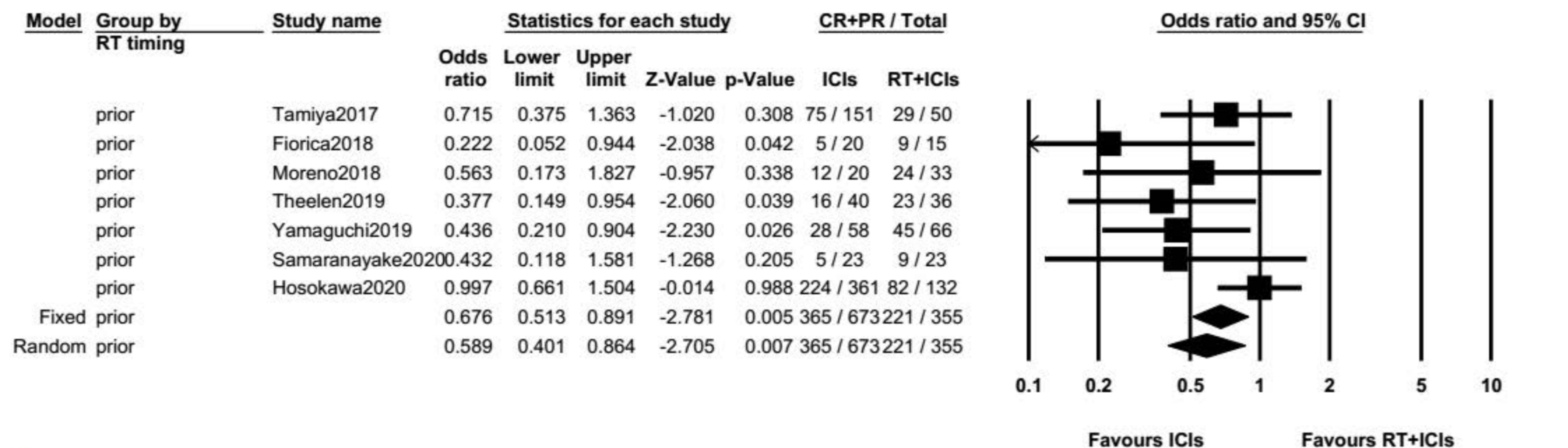

C

Supplement: Supplementary Materials — Supplemental Table 1: the PRISMA checklist. Supplemental Table 2: the example of search strategy using Embase database. Supplemental Table 3: quality assessment of included studies by NOS. Supplemental Table 4: the methodological quality evaluation of included studies by the Cochrane Handbook methods for RCTs. Supplemental Figure 1: meta-analysis of ORR in advanced NSCLC patients treated with ICIs versus RT + ICIs regimen. (A) The OR of advanced NSCLC patients in ICIs versus RT + ICIs group in the setting of study designs. The combined OR is in favor of RT + ICIs group. (B) The OR of ICIs versus RT + ICIs based on the disease condition. The improvement of ORR is in favor of RT plus ICIs. (C) The OR of ICIs versus RT + ICIs according to RT timing. Supplemental Figure 2: the ORR meta-analyses in terms of RT types and immunotherapy treatment line. (A) The impact of RT types on OR of ORR for ICIs versus ICIs + RT. (B) The impact of treatment line on OR of ORR for ICIs versus ICIs + RT. Supplemental Figure 3: meta-analysis results of DCR in ICIs versus RT + ICIs groups for advanced NSCLC patients. (A) Meta-analysis of DCR between ICIs and RT + ICIs groups in the setting of different study designs. (B) Subgroup meta-analysis of ICIs versus RT + ICIs with regard to study design. (C) Subgroup meta-analysis of patients from ICIs versus RT + ICIs groups based on RT timing. Supplemental Figure 4: the DCR meta-analyses in terms of RT types and immunotherapy treatment line. (A) The impact of RT types on OR of DCR for ICIs versus ICIs + RT. (B) The impact of treatment line on OR of DCR for ICIs versus ICIs + RT. Supplemental Figure 5: meta-analysis of PFS based on RT types in the concurrent RT group. Supplemental Figure 6: meta-analysis of OS based on RT BED. Supplemental Figure 7: the correlation analysis between BED and OS from RT + ICIs group. Supplemental Figure 8: ORR sensitivity analysis. Supplemental Figure 9: cumulative analysis of ORR. Supplemental Figure 10: funnel plot [file 4477263.f1.zip › Supplemental Figure 3 DCR.pdf]

A

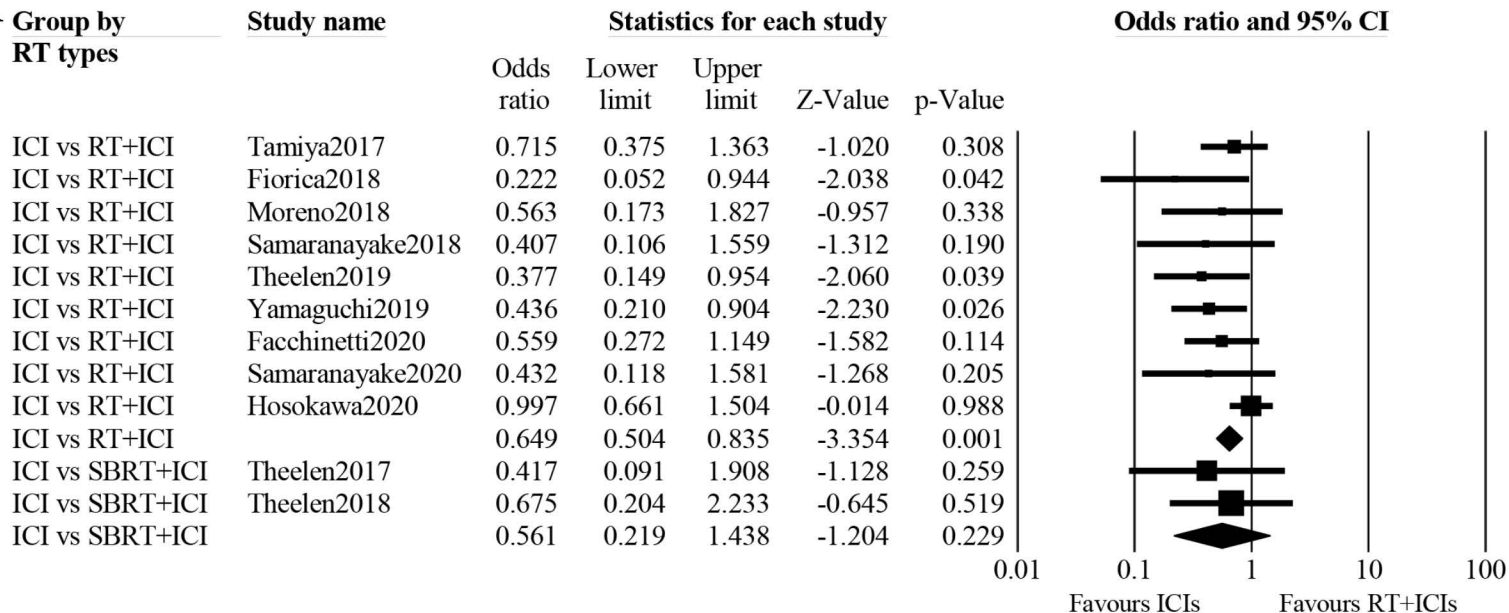

B

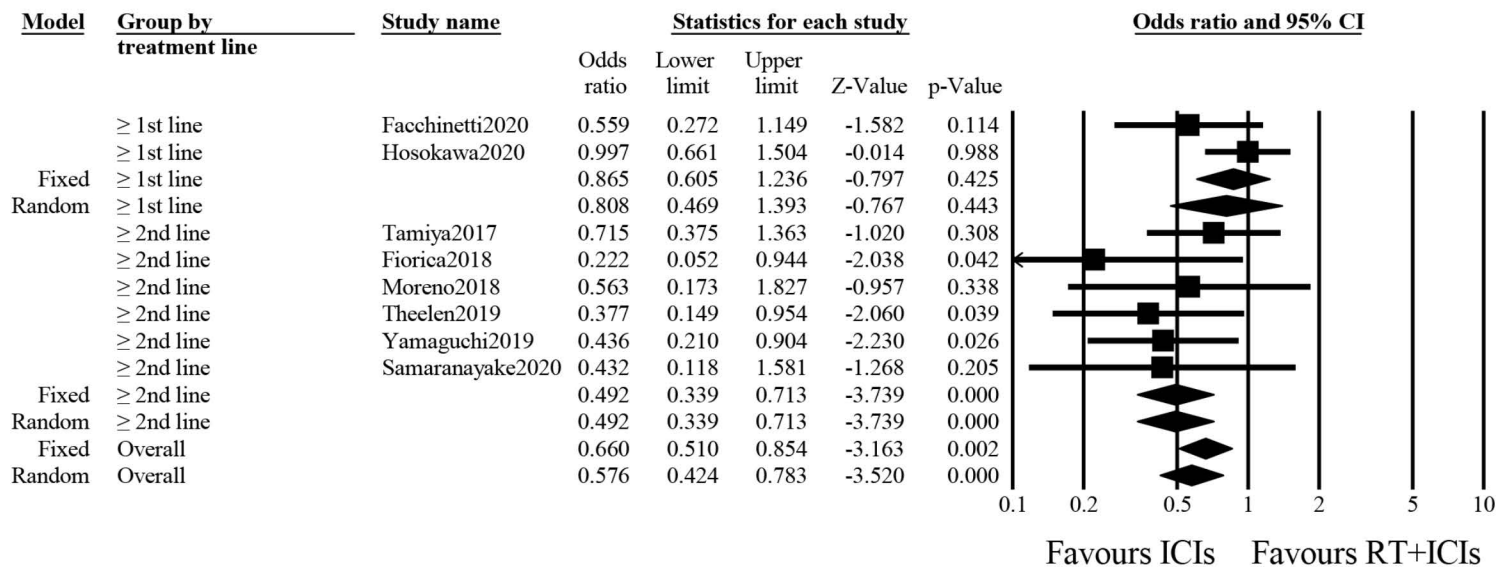

Supplement: Supplementary Materials — Supplemental Table 1: the PRISMA checklist. Supplemental Table 2: the example of search strategy using Embase database. Supplemental Table 3: quality assessment of included studies by NOS. Supplemental Table 4: the methodological quality evaluation of included studies by the Cochrane Handbook methods for RCTs. Supplemental Figure 1: meta-analysis of ORR in advanced NSCLC patients treated with ICIs versus RT + ICIs regimen. (A) The OR of advanced NSCLC patients in ICIs versus RT + ICIs group in the setting of study designs. The combined OR is in favor of RT + ICIs group. (B) The OR of ICIs versus RT + ICIs based on the disease condition. The improvement of ORR is in favor of RT plus ICIs. (C) The OR of ICIs versus RT + ICIs according to RT timing. Supplemental Figure 2: the ORR meta-analyses in terms of RT types and immunotherapy treatment line. (A) The impact of RT types on OR of ORR for ICIs versus ICIs + RT. (B) The impact of treatment line on OR of ORR for ICIs versus ICIs + RT. Supplemental Figure 3: meta-analysis results of DCR in ICIs versus RT + ICIs groups for advanced NSCLC patients. (A) Meta-analysis of DCR between ICIs and RT + ICIs groups in the setting of different study designs. (B) Subgroup meta-analysis of ICIs versus RT + ICIs with regard to study design. (C) Subgroup meta-analysis of patients from ICIs versus RT + ICIs groups based on RT timing. Supplemental Figure 4: the DCR meta-analyses in terms of RT types and immunotherapy treatment line. (A) The impact of RT types on OR of DCR for ICIs versus ICIs + RT. (B) The impact of treatment line on OR of DCR for ICIs versus ICIs + RT. Supplemental Figure 5: meta-analysis of PFS based on RT types in the concurrent RT group. Supplemental Figure 6: meta-analysis of OS based on RT BED. Supplemental Figure 7: the correlation analysis between BED and OS from RT + ICIs group. Supplemental Figure 8: ORR sensitivity analysis. Supplemental Figure 9: cumulative analysis of ORR. Supplemental Figure 10: funnel plot [file 4477263.f1.zip › Supplemental Figure 4 DCR updated 1 RT types and treatment line.pdf]

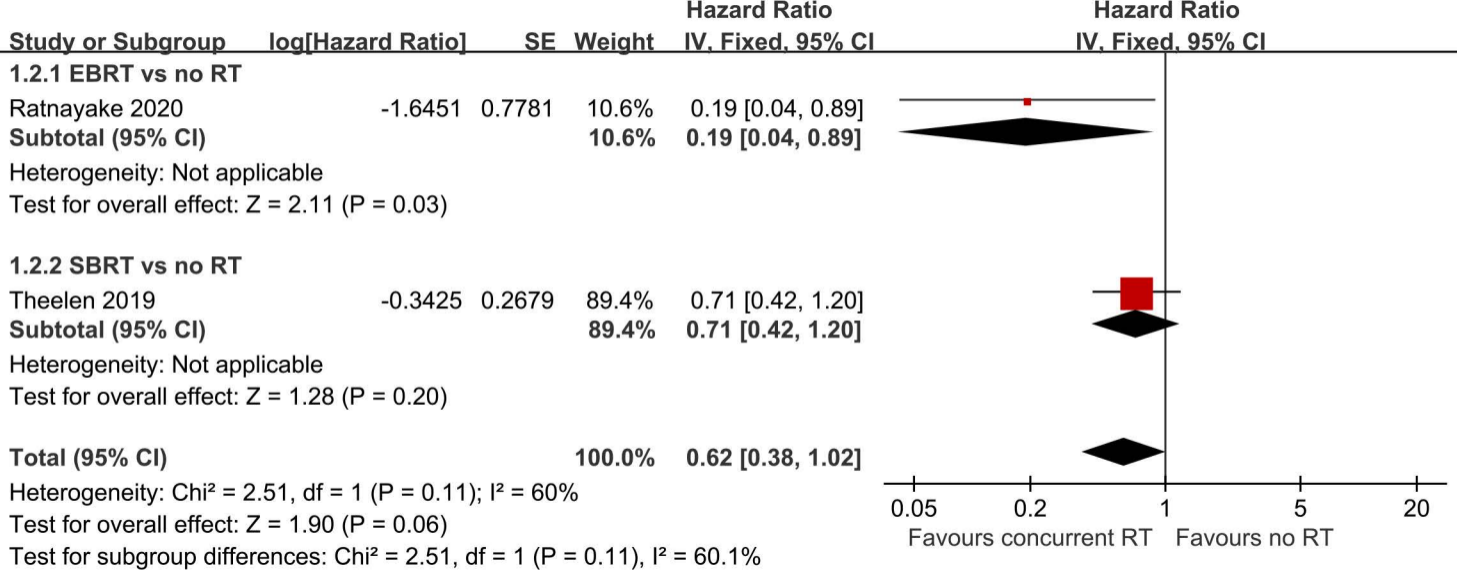

Supplement: Supplementary Materials — Supplemental Table 1: the PRISMA checklist. Supplemental Table 2: the example of search strategy using Embase database. Supplemental Table 3: quality assessment of included studies by NOS. Supplemental Table 4: the methodological quality evaluation of included studies by the Cochrane Handbook methods for RCTs. Supplemental Figure 1: meta-analysis of ORR in advanced NSCLC patients treated with ICIs versus RT + ICIs regimen. (A) The OR of advanced NSCLC patients in ICIs versus RT + ICIs group in the setting of study designs. The combined OR is in favor of RT + ICIs group. (B) The OR of ICIs versus RT + ICIs based on the disease condition. The improvement of ORR is in favor of RT plus ICIs. (C) The OR of ICIs versus RT + ICIs according to RT timing. Supplemental Figure 2: the ORR meta-analyses in terms of RT types and immunotherapy treatment line. (A) The impact of RT types on OR of ORR for ICIs versus ICIs + RT. (B) The impact of treatment line on OR of ORR for ICIs versus ICIs + RT. Supplemental Figure 3: meta-analysis results of DCR in ICIs versus RT + ICIs groups for advanced NSCLC patients. (A) Meta-analysis of DCR between ICIs and RT + ICIs groups in the setting of different study designs. (B) Subgroup meta-analysis of ICIs versus RT + ICIs with regard to study design. (C) Subgroup meta-analysis of patients from ICIs versus RT + ICIs groups based on RT timing. Supplemental Figure 4: the DCR meta-analyses in terms of RT types and immunotherapy treatment line. (A) The impact of RT types on OR of DCR for ICIs versus ICIs + RT. (B) The impact of treatment line on OR of DCR for ICIs versus ICIs + RT. Supplemental Figure 5: meta-analysis of PFS based on RT types in the concurrent RT group. Supplemental Figure 6: meta-analysis of OS based on RT BED. Supplemental Figure 7: the correlation analysis between BED and OS from RT + ICIs group. Supplemental Figure 8: ORR sensitivity analysis. Supplemental Figure 9: cumulative analysis of ORR. Supplemental Figure 10: funnel plot [file 4477263.f1.zip › Supplemental Figure 5 concurrent RT vs no RT for PFS.pdf]

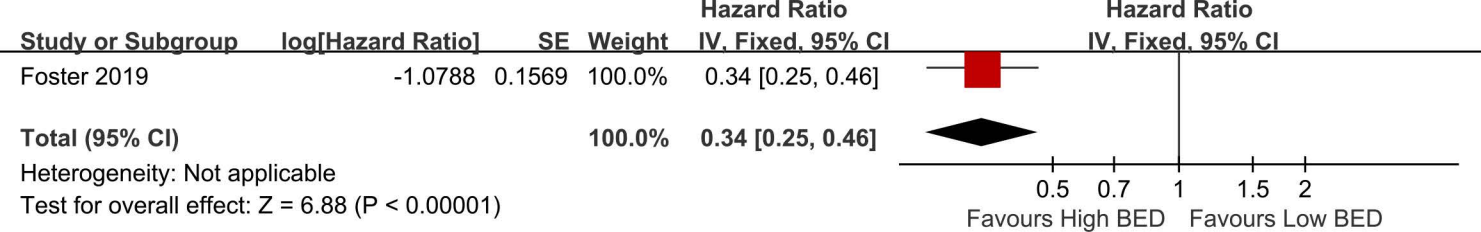

Supplement: Supplementary Materials — Supplemental Table 1: the PRISMA checklist. Supplemental Table 2: the example of search strategy using Embase database. Supplemental Table 3: quality assessment of included studies by NOS. Supplemental Table 4: the methodological quality evaluation of included studies by the Cochrane Handbook methods for RCTs. Supplemental Figure 1: meta-analysis of ORR in advanced NSCLC patients treated with ICIs versus RT + ICIs regimen. (A) The OR of advanced NSCLC patients in ICIs versus RT + ICIs group in the setting of study designs. The combined OR is in favor of RT + ICIs group. (B) The OR of ICIs versus RT + ICIs based on the disease condition. The improvement of ORR is in favor of RT plus ICIs. (C) The OR of ICIs versus RT + ICIs according to RT timing. Supplemental Figure 2: the ORR meta-analyses in terms of RT types and immunotherapy treatment line. (A) The impact of RT types on OR of ORR for ICIs versus ICIs + RT. (B) The impact of treatment line on OR of ORR for ICIs versus ICIs + RT. Supplemental Figure 3: meta-analysis results of DCR in ICIs versus RT + ICIs groups for advanced NSCLC patients. (A) Meta-analysis of DCR between ICIs and RT + ICIs groups in the setting of different study designs. (B) Subgroup meta-analysis of ICIs versus RT + ICIs with regard to study design. (C) Subgroup meta-analysis of patients from ICIs versus RT + ICIs groups based on RT timing. Supplemental Figure 4: the DCR meta-analyses in terms of RT types and immunotherapy treatment line. (A) The impact of RT types on OR of DCR for ICIs versus ICIs + RT. (B) The impact of treatment line on OR of DCR for ICIs versus ICIs + RT. Supplemental Figure 5: meta-analysis of PFS based on RT types in the concurrent RT group. Supplemental Figure 6: meta-analysis of OS based on RT BED. Supplemental Figure 7: the correlation analysis between BED and OS from RT + ICIs group. Supplemental Figure 8: ORR sensitivity analysis. Supplemental Figure 9: cumulative analysis of ORR. Supplemental Figure 10: funnel plot [file 4477263.f1.zip › Supplemental Figure 6 BED.pdf]

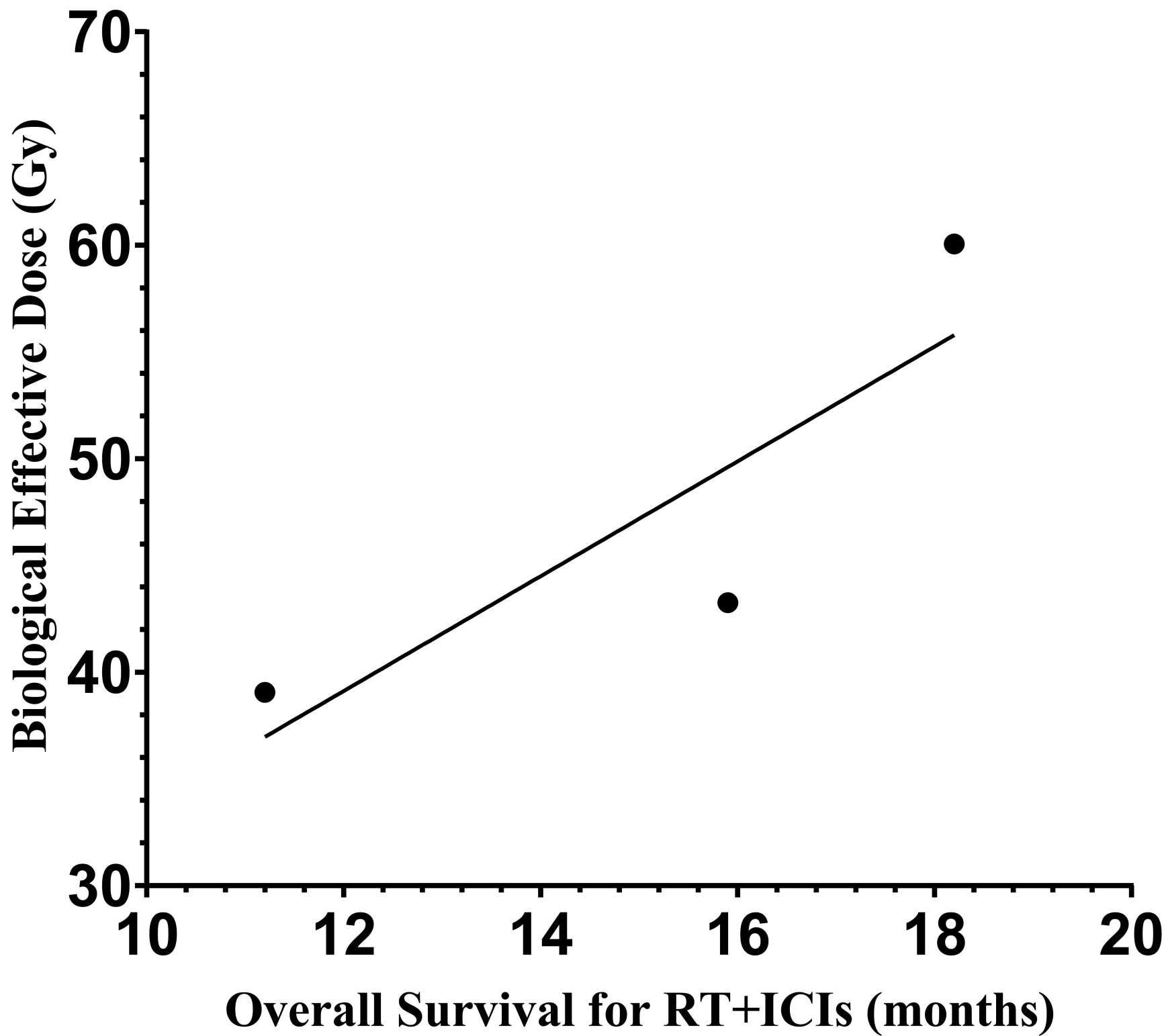

Supplement: Supplementary Materials — Supplemental Table 1: the PRISMA checklist. Supplemental Table 2: the example of search strategy using Embase database. Supplemental Table 3: quality assessment of included studies by NOS. Supplemental Table 4: the methodological quality evaluation of included studies by the Cochrane Handbook methods for RCTs. Supplemental Figure 1: meta-analysis of ORR in advanced NSCLC patients treated with ICIs versus RT + ICIs regimen. (A) The OR of advanced NSCLC patients in ICIs versus RT + ICIs group in the setting of study designs. The combined OR is in favor of RT + ICIs group. (B) The OR of ICIs versus RT + ICIs based on the disease condition. The improvement of ORR is in favor of RT plus ICIs. (C) The OR of ICIs versus RT + ICIs according to RT timing. Supplemental Figure 2: the ORR meta-analyses in terms of RT types and immunotherapy treatment line. (A) The impact of RT types on OR of ORR for ICIs versus ICIs + RT. (B) The impact of treatment line on OR of ORR for ICIs versus ICIs + RT. Supplemental Figure 3: meta-analysis results of DCR in ICIs versus RT + ICIs groups for advanced NSCLC patients. (A) Meta-analysis of DCR between ICIs and RT + ICIs groups in the setting of different study designs. (B) Subgroup meta-analysis of ICIs versus RT + ICIs with regard to study design. (C) Subgroup meta-analysis of patients from ICIs versus RT + ICIs groups based on RT timing. Supplemental Figure 4: the DCR meta-analyses in terms of RT types and immunotherapy treatment line. (A) The impact of RT types on OR of DCR for ICIs versus ICIs + RT. (B) The impact of treatment line on OR of DCR for ICIs versus ICIs + RT. Supplemental Figure 5: meta-analysis of PFS based on RT types in the concurrent RT group. Supplemental Figure 6: meta-analysis of OS based on RT BED. Supplemental Figure 7: the correlation analysis between BED and OS from RT + ICIs group. Supplemental Figure 8: ORR sensitivity analysis. Supplemental Figure 9: cumulative analysis of ORR. Supplemental Figure 10: funnel plot [file 4477263.f1.zip › Supplemental Figure 7 Correlation of BED and OS.pdf]
